# Host identity, more than elevation, shapes bee microbiomes along a tropical elevation gradient

**DOI:** 10.3389/fmicb.2025.1671348

**Published:** 2025-09-18

**Authors:** Andrea Pinos, Pedro Alonso-Alonso, Yenny Correa-Carmona, Kim L. Holzmann, Felipe Yon, Gunnar Brehm, Ingolf Steffan-Dewenter, Marcell K. Peters, Arne Weinhold, Alexander Keller

**Affiliations:** ^1^Cellular and Organismic Networks, Faculty of Biology, Ludwig-Maximilians-Universität München, Martinsried, Germany; ^2^Department of Animal Ecology and Tropical Biology, Biocenter, University of Würzburg, Würzburg, Germany; ^3^Institut für Zoologie und Evolutionsbiologie mit Phyletischem Museum, Friedrich-Schiller University Jena, Jena, Germany; ^4^Departamento de Ciencias Biológicas y Fisiológicas, Facultad de Ciencias e Ingeniería, Universidad Peruana Cayetano Heredia, Lima, Peru; ^5^Instituto de Medicina Tropical, Universidad Peruana Cayetano Heredia, Lima, Peru

**Keywords:** environmental gradient, host-microbiome interactions, microbial diversity, bee tribes, host-taxonomic identity, gut microbial communities, Andean-Amazonian forests

## Abstract

Understanding how host-microbiome interactions respond to abiotic and biotic factors is key to elucidating the mechanisms influencing ecological communities under current climate change scenarios. Despite increasing evidence that gut microbial communities associated with bees influence their health and fitness, including key roles in nutrient assimilation, toxin removal, defense against pathogens, and immune responses, the distribution of gut microbial communities and the dynamics of these associations along environmental gradients remain poorly understood. In this study, we assessed how environmental changes with elevation and host taxonomic identity influence the bacterial gut microbiome of wild bees collected along a 3,600 m elevation gradient in the Peruvian Andes. We applied DNA metabarcoding on the 16S rRNA region of gut samples from five bee tribes: Apini (honey bees), Bombini (bumble bees), Meliponini (stingless bees), Euglossini (orchid bees), and Halictini (sweat bees). Our findings indicate a general decrease in bacterial diversity and a high turnover of microbial taxa along the elevation gradient, with notable differences among host tribes. Host taxonomic identity was a strong predictor of gut microbial community composition, despite a high turnover of microbial and host taxa along the gradient. Within tribes, the turnover of microbial compositions was mainly explained by environmental changes with elevation in bumble and stingless bees. The observed variations in gut microbial diversity and composition at different elevations and different host taxa suggest that both factors significantly impact the gut microbiomes. As climate change continues to influence environmental conditions in the Andean-Amazonian forests it is crucial to consider how these changes may affect host-microbiome relationships. This highlights the necessity of understanding both abiotic and biotic factors in the context of climate change.

## 1 Introduction

Environmental conditions shape diversity patterns, community composition, and biotic interactions in natural ecosystems (Nottingham et al., 2018; Peters et al., 2019). Consequently, climate change with increasing temperatures is expected to restructure trophic interactions and impact ecosystem dynamics (McCain and Garfinkel, 2021; Yuan et al., 2021). Changes in ecological properties, such as species diversity, phenology, or community composition, are known to affect ecosystem functioning and stability (Dzekashu et al., 2023; Hoiss et al., 2015; König et al., 2022; Lara-Romero et al., 2019). However, unraveling interspecific interactions is necessary for understanding species coexistence and predicting ecological responses under future climate change scenarios (Hoiss et al., 2015; Yuan et al., 2021).

Mountain ecosystems provide ideal natural laboratories to study the effects of global warming on a smaller spatial scale (Wang et al., 2022). These environmental gradients are characterized by shifts in abiotic conditions, in particular shifts in temperature and precipitation, resulting in a turnover of species across relatively short geographic distances (Bryant et al., 2008; Siles and Margesin, 2016). As a result, elevational gradients have been widely used to examine the responses of various taxonomic groups to environmental change (Dzekashu et al., 2022; Hoiss et al., 2015; Perillo et al., 2021; Peters et al., 2016; Pitteloud et al., 2021). A well-established pattern is that the species richness of most above-ground organisms tends to decline with elevation or peak at mid-elevations (Peters et al., 2016). The metabolic theory is frequently invoked to explain this trend, positing that ecological and evolutionary rates in ectothermic organisms, are tied to ambient temperature (Brown et al., 2004; McCain and Grytnes, 2010). This suggests that varying rates of evolution and biotic interactions occurring along climatic gradients on mountains influence diversity gradients (Pellissier et al., 2018; Yuan et al., 2021).

It remains largely unclear how patterns and drivers of species diversity can be transferred to microbial communities within insect guts. Research on microbial diversity across elevation gradients has focused mainly on soil or plant-associated communities. For instance, Bryant et al. (2008) found that soil bacterial richness consistently declined from the lowest to the highest elevations in the Rocky Mountains of Colorado, whereas Nottingham et al. (2018) observed a similar decline in soil microbial richness along an elevation gradient in the Andean mountains. Fierer et al. (2011) reported that bacterial diversity in organic soil, mineral soil, as well as on leaf surfaces exhibited no significant elevation pattern, which stands in strong contrast to the clear diversity changes observed in plant and animal communities along the same elevation gradient in eastern Peru. For fungi, no significant differences were found in plant endophyte diversity along an elevation gradient in Mauna Loa on Hawai‘i (Cobian et al., 2019). These results highlight a key knowledge gap in ecology about microbial diversity gradients: Despite their ubiquity, abundance, and functional importance, microbes may follow distinct biogeographical rules that are not adequately explained by theories developed for macro-organisms (Fierer et al., 2011). As such, inclusive and revised conceptual frameworks are needed to account for microbial dynamics, particularly within host-associated systems like the animal gut.

There is growing evidence that the gut microbial communities associated with bees influence their health and fitness (Engel et al., 2016; Li et al., 2023). Gut symbionts play a key role in nutrient assimilation, toxin removal, defense against pathogens, and the immune responses (Douglas, 2015; Schmidt and Engel, 2021) and can modulate the physiology and behavior of their bee hosts (Zheng et al., 2018). Gut microbes associated with bees are transmitted through two distinct routes: First, through social interactions with conspecifics (i.e., vertically transmitted), allowing these symbionts to be passed on for generations (Mee and Barribeau, 2023). Alternatively, they can be acquired from the surrounding environment (i.e., horizontally transmitted) as short-term residents in the gut (Mayr et al., 2021; Voulgari-Kokota et al., 2019). The transmission routes of gut microbes vary among the bee taxa due to their distinct lifestyles and social structures (Mee and Barribeau, 2023). For instance, social bees like honey bees, bumble bees and stingless bees often engage in frequent physical interactions, facilitating vertical transmission of microbes through social grooming and trophallaxis (Hammer et al., 2021b; Zheng et al., 2018). In contrast, orchid and sweat bees may rely more on horizontal transmission from their environment, as their social interactions are less frequent or more opportunistic (Mee and Barribeau, 2023; Voulgari-Kokota et al., 2019). As a consequence, these associations range from highly host-specialized gut microbial communities (Koch et al., 2013; Kwong et al., 2017; Zheng et al., 2018), to those hosts with environmentally driven microbial communities (Keller et al., 2021; McFrederick et al., 2012; Voulgari-Kokota et al., 2019).

Insect-associated microbial communities, especially those of bees, provide an excellent opportunity to explore host-microbiome dynamics across environmental gradients. In general, gut microbial communities are fundamentally distinct from environmental microbiomes, due to the unique biotic and abiotic conditions within host organisms (Thompson et al., 2017). Considering the important interactions between the gut symbionts, their hosts, and the environment, it is essential to unravel the factors that shape the microbial community structures in wild pollinators (Li et al., 2023). A key question is whether microbial communities of ectothermic bee hosts are more strongly shaped by host-taxonomic identity or by environmental factors. To date, only a few studies have reported patterns of gut microbiomes associated with bees along elevation gradients. For instance, Dong et al. (2024) reported species replacement in both gut symbionts and bumble bees with increasing elevation in a gradient in Hengduan Mountains of southwestern China. Mayr et al. (2021) reported the highest gut bacterial diversity and turnover of microbial taxa associated with *Lasioglossum* bees at high elevations across an elevation gradient at Mt. Kilimanjaro, Tanzania. Conversely, in honey bees the highest microbial diversity was found at lower elevations in a gradient in Tamil Nadu, India, with microbial taxa dynamically shifting, suggesting potential adaptations of gut microbiota to different ecological niches (Hariprasath et al., 2025). However, these studies are restricted each to a single bee group, which limits the understanding of how different hosts might influence gut microbial communities along the same environmental gradient.

In this study, we address this limitation by examining the five bee tribes from the corbiculate bees, i.e., Apini (honey bees), Bombini (bumble bees), Meliponini (stingless bees), Euglossini (orchid bees), and the Halictini (sweat bees) tribe, all of them differing in phylogeny, ecology, and thermoregulatory capacity. Bees are not strictly ectothermic; many exhibit facultative thermoregulation, actively modulating their colony or body temperature and, by extension, the gut environment (Hammer et al., 2021a). Nonetheless, the plasticity of thermoregulation varies throughout bee phylogeny, and among individuals, across time, and location. As a consequence, some bee species are exposed to more significant fluctuations in temperature than others, which can influence the interactions with their gut microbial symbionts (Hammer et al., 2021a).

Understanding how elevation and host taxonomy influence the diversity and composition of gut microbial communities is essential for predicting how mutualistic interactions will respond to climate change (Engel et al., 2016; Pellissier et al., 2018). To our knowledge, no previous study has investigated these dynamics across an entire elevation gradient (covering tropical lowland, mountain and cloud forests up to the tree line) with 29 bee genera across five bee tribes encompassing a broad taxonomic range. Here, we assess the diversity and composition patterns of gut microbial communities across a ~3600-m elevation range in the Peruvian Andes, using 16S rRNA gene metabarcoding. Our research aims to answer the following questions:

1. How does bacterial diversity of bee microbiomes vary with elevation; does it decrease linearly, or does it peak at mid-elevations along the elevation gradient?
2. Does host taxonomy and elevation jointly or independently influence the variation in microbial community composition?
3. Does the composition of gut microbial communities change along the elevation gradient and between host tribes?

Our study offers novel insights into how host identity and environmental conditions interact to shape bee-microbiome associations along an elevation gradient, with implications for better understanding bee health and resilience under climate change.

## 2 Methods

### 2.1 Study area

**Figure 1:**
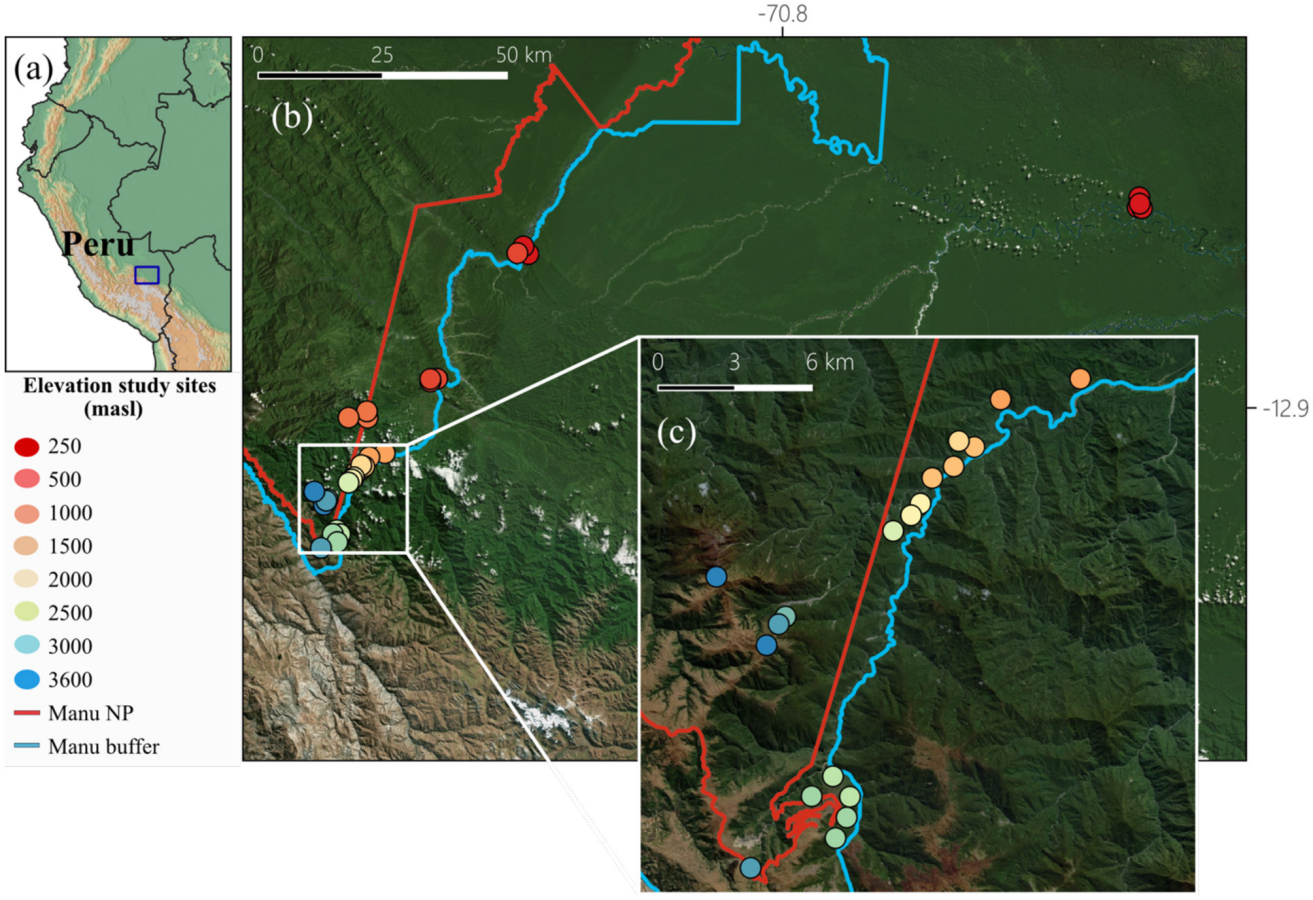
**(a)** Map of the study area along the Kosñipata valley in Peru, **(b)** locations and elevations of the 33 study sites from which the microbial communities of the bees were determined. The Manu buffer line indicates the transition between the protected area Manu NP and surrounding landscapes. Colors of dots indicate plot elevations. **(c)** Zoomed- in view of the high and mid elevations of the gradient (map lines delineate study areas in the National Park Manu and do not necessarily depict accepted national boundaries). Map created using Bing Maps. Microsoft product screen shot(s) reprinted with permission from Microsoft Corporation. For more information, visit Microsoft Print Rights.

The study was conducted in the Kosñipata valley in south-eastern Peru. This region offers access to largely intact forest habitats along a complete elevation gradient. Consequently, many different habitat types are present to cover tropical lowlands, mountain rainforests at mid-elevations, and vegetation in the highlands. Our research was conducted on 33 collection sites in the forest and around the research stations and camps (Tres Cruces, Wayqecha, San Pedro, Tono, Pantiacolla, Villa Carmen, Los Amigos) from 245 m above sea level (masl) to 3,658 masl (Figure 1; Supplementary Table S1). The temperature was recorded by one TMS logger and one iButton per plot, which took a reading every 15 min in 4 h intervals, respectively, for a complete year (September 2022–December 2023; Supplementary Table S1), for details see Holzmann et al. (2025). Samples were collected between September to December 2022, i.e., in a period with less than the average precipitation.

### 2.2 Bee sampling and specimen identification

We targeted to collect at least *N* = 3 samples for each bee species per study site. Bees were collected manually by net sweeping and placed in sterile collection tubes with 96% ethanol. Collected bees were stored at the research stations/base camps in an insulated cooler with ice packs until being dissected. Each bee was individually dissected on plastic petri dish using a scalpel and dissection scissors (all sterilized) to obtain the entire gut region. Gut samples were stored in 96 well plates filled with Zymo DNA/RNA shield lysis solution to preserve nucleic acid until isolation in the lab.

All collected bees were allocated to the different taxonomic groups morphologically in the field as accurately as possible according to relevant reference materials (Allen and Villacampa, 2017; Fernández and Sharkey, 2006; Rasmussen, 2003; Rasmussen and Vasquez, 2019). Additionally, we removed one leg from each bee for further confirmation of species identity by DNA barcoding. Taxonomic identification of bees by DNA barcoding was performed at the Canadian Center for DNA Barcoding (CCDB; Guelph, Ontario, Canada; http://ccdb.ca/). COI sequences were submitted to the Barcode of Life Database (http://www.boldsystems.org) for taxonomic assignment. A phylogenetic analysis of each tribe was conducted to link the morphological and molecular classifications. The sequences were aligned using MUSCLE (Edgar, 2004), and the final alignment was > 600 bp long. Reference sequences were obtained by a BLAST search against GenBank (Benson et al., 2014). We selected closely related taxa as well as outgroup sequences to construct a neighbor-joining tree using MEGA version 11.0.13 (Hall, 2013).

### 2.3 Microbiome profiling by DNA metabarcoding

The genomic DNA from the guts was isolated using the Zymo BIOMICS™ 96 DNA kit for microbiome or metagenome analyses, according to manufacturer instructions (Zymo Research, D4309). Each plate contained 88 samples and was accompanied by at least four negative extraction controls (water) and four positive extraction controls (ZymoBiomics Mock community). The concentration of the isolated DNAs was quantified using a Qubit^®^ 4.0 Fluorometer (Invitrogen, Carlsbad, CA, USA). 16S rRNA gene libraries were constructed using a dual-indexing approach as described in Kozich et al. (2013) to amplify the V4 variable region of the 16S rRNA gene. We used 16 forward primer with index sequences SA501–SB508 and 24 reverse primer with indices SA701–SB712, allowing a total of 384 unique combinations for sample indexing (Additional file of Kozich et al.). PCR was performed in triplicates following the 96-well PCR sample design two as described in Sickel et al. (2015; Additional file of Sickel et al.). To avoid bias triplicate reactions of each sample were combined after PCR and further processed as described in Kozich et al. (2013). To pool each 96-well plate sample, the normalization was performed using the SequalPrep™ Normalization Plate Kit (Invitrogen) and concentrated using AMPure XP beads. Pools were quality controlled using a Bioanalyzer High Sensitivity DNA Chip (Agilent Technologies, Santa Clara, CA, USA), quantified with the dsDNA High Sensitivity Assay using a Qubit^®^ 2.0 Fluorometer (Invitrogen, Carlsbad, CA, USA). Then, pools were combined into a single pool and afterward diluted to have an equimolar concentration of 1.8 nM. Eight pM of the libraries combined with 5% Phix Control (Illumina Inc., San Diego, CA, USA) was sequenced with the Illumina MiSeq platform at The Center for Genome Studies, LMU Biozentrum using 2 × 250 cycles reagent kit v2 according to the manufacturer's instructions (Illumina Inc., San Diego, USA).

### 2.4 Bioinformatic analysis and data availability

Raw sequence reads were obtained directly from the Illumina MiSeq output, which includes sample reads already demultiplexed by MID barcode with the Illumina device's software. Bioinformatic analysis followed the pipeline established by Leonhardt et al. (2022), available at https://github.com/chiras/metabarcoding_pipeline. Low-quality (E_max_ = 1, no ambiguous base pairs) and short (<170 bp) reads were removed. Cleaning of reads, i.e., removal of bad quality reads and filtering of chimeric artifacts, denoising, and dereplication to amplicon sequence variants (ASVs) was performed with VSEARCH global alignments (Rognes et al., 2016). The taxonomy assignment was made using direct global alignment classifications against the databases RDP_16s (v18; Edgar, 2016), GreenGenes 16s (v13.5; DeSantis et al., 2006), and SILVA 16S (v123; Quast et al., 2012) using VSEARCH and a threshold of 97% identity. Remaining unclassified reads were hierarchically classified using the SINTAX implementation of VSEARCH against SILVA 16S (v123) using a threshold cutoff of 0.8 (Edgar, 2016). Microbiome data was imported into R (R Core Team, 2021) and managed with the phyloseq package (McMurdie and Holmes, 2013). Positive controls were removed from the data set to mitigate any potential spillover into the samples. Any chloroplast or mitochondrial ASVs were identified in the ASV table and excluded.

### 2.5 Data analyses

All analyses were performed in R version 4.4.1 (R Core Team, 2021) using the following packages: Vegan (Oksanen et al., 2013), iNEXT (Hsieh et al., 2016), Betapart (Baselga and Orme, 2012), lme4 (Bates, 2010), r2glmm (Jaeger, 2017), MuMIn (Barton, 2009), and Microbiome (Lahti and Shetty, 2017).

#### 2.5.1 Covariation of elevation and temperature

Considering that temperature can influence the patterns of biotic variables along the elevation gradient (Peters et al., 2019), we assessed for all subsequent analyses whether temperature might be a more robust predictor of the observed variations in microbial community than elevation. We calculated the average temperature recorded by the data loggers per each plot. We compared models using Akaike's Information Criterion with the *model.sel* function (Barton, 2009). The goodness of fit between alternative models was tested with an ANOVA. Since both variables were highly correlated, they appeared to be mostly interchangeable in our study. Elevation was, in most cases, slightly more predictive; therefore, we used this as the explanatory variable in the following analyses.

#### 2.5.2 Effect of elevation on microbial richness and Shannon diversity (α-diversity)

Estimates of alpha diversity (local species richness and diversity) were analyzed using the iNEXT approach, which applies rarefaction and extrapolation to standardize the variables for sampling effort (Hsieh et al., 2016). We calculated microbial species richness (*q* = 0; thereafter called “microbial richness”) and microbial species diversity (*q* = 1, i.e., the Shannon diversity index; thereafter called “microbial diversity”). For each host tribe, differences among microbial richness and diversity with elevation were determined using a negative binomial generalized linear models (GLMs) and corresponding 95% confidence intervals (95% CI) with alpha diversity indices as the response variable and elevation (continuous) as the predictor variable. The relationships between microbial richness and diversity with elevation, were explored with linear and quadratic models. The better model was selected based on lower value of Akaike's information criterion (Yamaoka et al., 1978).

#### 2.5.3 Effects of elevation and host identity on bacterial community composition (β-diversity)

To characterize differences in microbial community composition among the different host tribes along the elevation gradient, we estimated overall community beta-diversity with the Bray-Curtis dissimilarity index (Bray and Curtis, 1957) using ASV abundances.

Compositional changes along the elevation gradient were investigated by examining the correlation between elevation and community compositional dissimilarities using Mantel tests with 10,000 permutations (Mantel, 1967).

Next, to investigate and visualize correlations among the explanatory variables influencing microbial community composition, we applied non-metric multidimensional scaling (NMDS) along two axes based on Bray-Curtis dissimilarity. A permutational multivariate analysis of variance model (PERMANOVA, adonis2) was performed on the Bray-Curtis dissimilarity matrix by applying the *adonis2* function to discern the amount of variation attributed to elevation and host tribe (with 999 permutations). Our models included host taxon at different levels: species level for bumble bees, while for stingless bees, orchid bees, and sweat bees, it was assessed at the genus level. For honey bees, the host genus or species could not be utilized as a variable due to the sampling of only one species within this group. *R*^2^ values were obtained using the Nakagawa and Schielzeth approach (Nakagawa and Schielzeth, 2013).

#### 2.5.4 Variance partitioning

The relative contributions of the elevation and host identity in shaping microbial community composition were estimated by variance partitioning analysis using the *varpart* function. For this method, forward selection based on redundancy analysis (RDA) of variables was conducted to exclude the variables that did not significantly explain patterns of community similarity. We started from intercept-only and all-variables were modeled with the *ordiR2step* function. The respective amounts of variance (i.e., individual and shared) were determined by the adjusted *R*^2^ with redundancy analysis (RDA; Ramette and Tiedje, 2007). An ANOVA test was carried out to test whether there was a significant difference in the individual contribution of elevation and host tribe on the microbial composition.

#### 2.5.5 Partitioning of β-diversity components (turnover and nestedness)

To further quantify the variations in beta diversity along our gradient, we analyzed the relative contributions of species nestedness and species turnover for each host separately (Baselga, 2017) using the *beta.multi.abund* function (Baselga and Orme, 2012). The component β_BC − bal_ reflects changes in community composition resulting from balanced variations in abundance, where individuals of specific species at one site are replaced by an equivalent number of individuals from different species at another site (i.e., species turnover; Baselga, 2017). The component β_BC − gra_ represents changes in community composition driven by abundance gradients, where some individuals are lost from one site to another (i.e., community nestedness; Baselga, 2017). Then we performed Mantel tests to analyze the correlation between each beta diversity component (i.e., turnover and nestedness) and elevation.

#### 2.5.6 Elevation effects on gut microbial dominant bacterial groups

To assess whether the environmental gradient and host identity lead the turnover of microbial communities, we analyzed the changes in relative abundances of the most abundant bacterial families present in the gut of most of the organisms along the elevation gradient. This included the *Acetobacteraceae* and *Neisseriaceae* families from the phylum Proteobacteria, as well as the *Lactobacillaceae* and *Orbaceae* families from the phylum Firmicutes. The relationships between microbial relative abundances and elevation were tested using generalized linear models (GLMs), with the data family set to “Gaussian” and corresponding 95% confidence intervals (95% CI). The relationships between the relative abundances and elevation were explored with linear and quadratic models. The better models were selected based on lower value of Akaike's information criterion (Yamaoka et al., 1978).

#### 2.5.7 Core microbiome

To determine the consistent presence of microbial taxa across our five hosts tribes, we identified the “core microbiome.” Core taxa at genus level were defined as those present at a relative abundance of above 1% and a prevalence of 50% for each host tribe.

## 3 Results

### 3.1 Host distribution along the elevation gradient

We collected a total of 820 individuals from five bee tribes: honey bees (only *Apis mellifera, n* = 74) ranging from 275 to 3,000 masl; bumble bees (*n* = 44, five *Bombus* species) found between 275 and 3,658 masl; stingless bees (*n* = 393, representing 16 genera) occurring between 245 and 3,000 masl; orchid bees (*n* = 152, from four genera) distributed between 245 and 2,250 masl; and sweat bees (*n* = 157, consisting of 11 genera) also found between 245 and 3,000 masl (Supplementary Figure S1; Supplementary Table S2). Our barcoding approach confirmed these identifications, although stingless, orchid, and sweat bees could not be identified at species level.

We observed distinct elevational patterns in the distribution of bee taxa. Bumble bee species were present throughout the entire elevation gradient but were more concentrated either at high or low elevations. In contrast, stingless and sweat bees reached up to 3,000 masl, exhibiting the highest richness at low and mid-elevations. Orchid bees were found up to 2,250 masl, with their highest richness occurring at low elevations (Supplementary Figure S2; Supplementary Table S2).

### 3.2 Microbial richness and diversity are influenced by elevation

**Figure 2:**
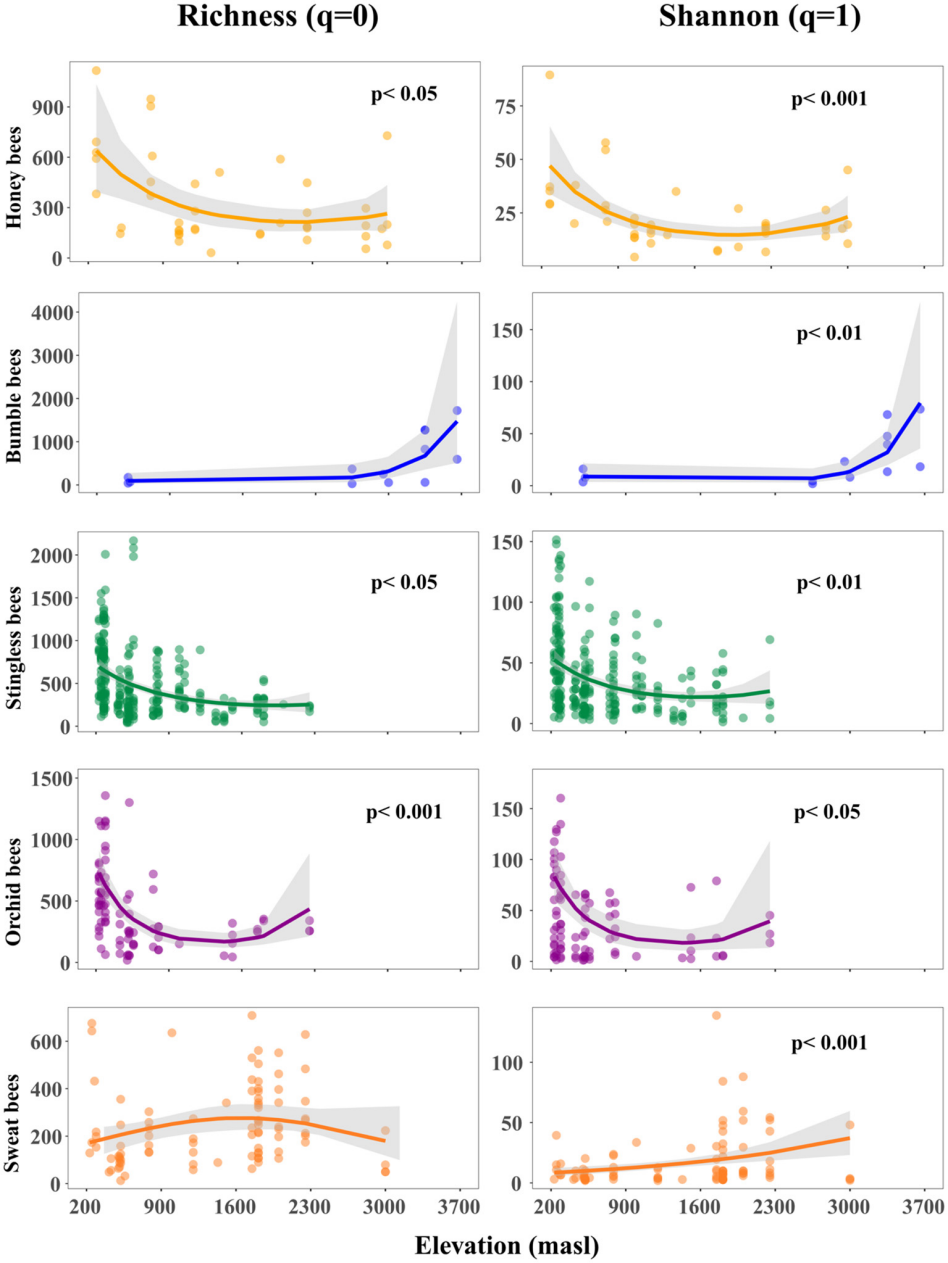
Effect of elevation on gut microbial richness and diversity arranged by host tribe along the elevation gradient. Points represent sampled bees at each study site. The trend lines along elevation were modeled with binomial negative generalized linear models and indicate the slope estimate. The gray shadows are the 95% confidence intervals around the estimate. The better fit model between linear and quadratic was selected based on the lower value of Akaike's information criterion.

Our results indicated that elevation has a more pronounced and variable impact on microbial richness and diversity across different bee host tribes compared to temperature, suggesting that elevation is a more informative variable for understanding the dynamics of microbial communities in this context (Supplementary Table S3). Our findings indicated a non-linear relationship between elevation with microbial richness and diversity within honey-, stingless- and orchid bees (Figure 2, Supplementary Table S3). We observed an initial decline followed by a slight increase at higher elevations. In contrast, we found a positive non-linear relationship between elevation and microbial diversity in bumble bees. For sweat bees, microbial richness exhibited an unimodal pattern that was marginally significant, with the highest richness observed at mid elevations, while microbial diversity increased linearly with elevation.

### 3.3 Elevation and host taxonomic identity both predict bacterial community composition

The Mantel test revealed that elevation influences microbial community structure; thus, bees located at similar elevations tend to have more similar microbial communities than bees from differing elevations (Table 1a). In four of our five host tribes (bumble-, stingless-, orchid-, and sweat bees) elevation correlated with dissimilarities in gut microbiome compositions. In contrast, elevation had no influence on the microbial beta diversity in honey bees (Table 1a). This highlights a more pronounced effect of elevation on microbial community composition in the four native host tribes.

Table 1Variation in microbial community composition (beta-diversity) among host tribes along the elevation gradient using Bray-Curtis distance matrices determined by **(a)** Mantel correlations and **(b)** PERMANOVA.

**Table:** 

**(a) Host**	**Predictor**	**Mantel**	
		***r***	***p-*** **value**
All hosts	Elevation	0.04	0.01^*^
Honey bees		0.03	0.25
Bumble bees		0.2	<0.001^***^
Stingless bees		0.04	0.01^*^
Orchid bees		0.14	<0.001^***^
Sweat bees		0.24	<0.001^***^

**Table:** 

**(b) Host**	**Predictor**	**PERMANOVA**		
		**R** ^2^	**F**	***p-*** **value**
All taxa	Full model	0.1	17.23	<0.001^***^
	Elevation	0.01	11.61	<0.001^***^
	Host tribe	0.09	19.86	<0.001^***^
Honey bees	Elevation	0.02	1.65	0.04^*^
Bumble bees	Full model	0.32	3.62	<0.001^***^
	Elevation	0.11	4.9	<0.001^***^
	Species	0.29	3.98	<0.001^***^
Stingless bees	Full model	0.14	3.86	<0.001^***^
	Elevation	0.01	4.41	<0.001^***^
	Genus	0.13	3.86	<0.001^***^
Orchid bees	Full model	0.07	2.56	<0.001^***^
	Elevation	0.03	5.08	<0.001^***^
	Genus	0.04	1.91	0.002^**^
Sweat bees	Full model	0.17	2.81	<0.001^***^
	Elevation	0.04	5.97	<0.001^***^
	Genus	0.15	2.69	<0.001^***^

Significant differences are denoted with asterisks as follows: ^*^p < 0.05, ^**^p < 0.01, and ^***^p < 0.001.

**Figure 3:**
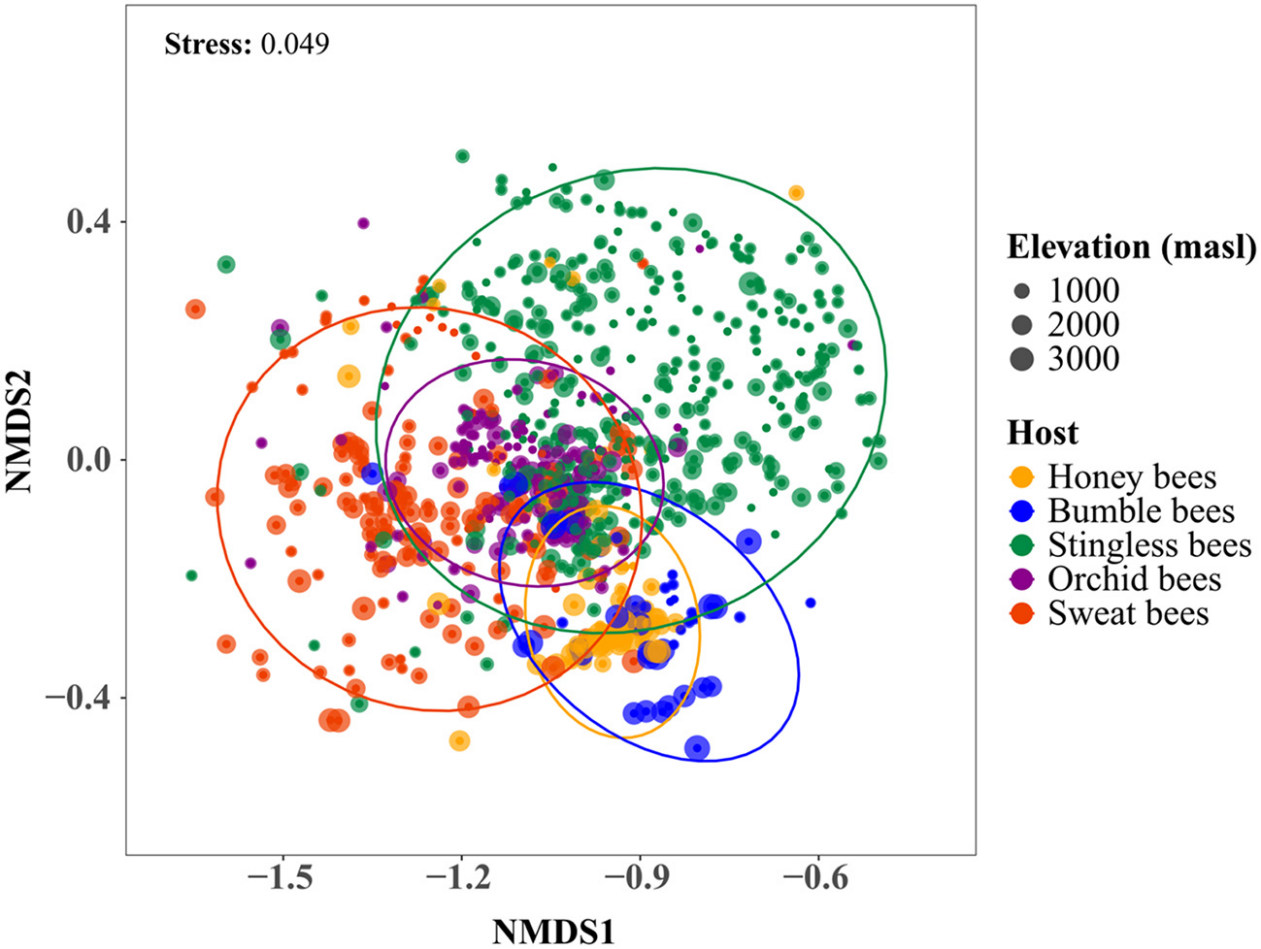
Bee gut microbial communities ordinated by non-metric multidimensional scaling (NMDS) based on Bray-Curtis dissimilarities. Each point represents an individual bee sample. Colors indicate different bee tribes. Different sized dots represent samples from different elevations. Stress value represents the goodness of fit. Ellipses indicate the 95% confidence interval around the centroid location of the data points, representing standard deviation.

The NMDS indicated that the differences in the microbial community composition were associated with the host tribe (Figure 3). A PERMANOVA revealed that 9% of the dissimilarity in the gut bacterial communities was attributed to host tribe, while elevation accounted for only 1% (Table 1b). When examining host tribes individually, the variation in microbial community composition explained by the host taxonomic identity was higher than that explained by elevation, although both factors accounted for a small portion of the total variation (Table 1b).

The variance partitioning results indicated that 8% of the total variance can be attributed to the host identity, while elevation explained 4% (Table 2). This relationship accounted for a relatively small but significantly correlated effect as predictors of variation in microbial community composition when host tribes were considered together. These results were further examined by analyzing host tribes separately; for both bumble bees and sweat bees, the host species and genera was the best predictor of community composition compared to elevation, respectively (Table 2). Interestingly, the largest portion of the explained variation in community structure (14%) for bumble bees was attributed to the shared fraction between elevation and host species. This finding indicates that the influences of elevation and host species on bumble bee community structure cannot be separated independently due to their significant interaction effect. On the other hand, elevation accounted for the highest variation in microbial community structure for stingless and orchid bees (Table 2).

**Table 2:** Variance partitioning analysis.

**Host**	**Predictor**	**Adjusted *R*^2^**	***F***	***p-*value**
All hosts	Full model	0.14	5.15	<0.001^***^
	Overlap	0.02	–	–
	Elevation	0.04	2.91	<0.001^***^
	Host tribe	0.08	24.42	<0.001^***^
Bumble bees	Full model	0.17	1.67	0.01^*^
	Overlap	0.14	-	-
	Elevation	0.01	1.76	0.02^*^
	Species	0.02	3	<0.001^***^
Stingless bees	Full model	0.14	2.72	<0.001^***^
	Overlap	0.01	–	–
	Elevation	0.07	2.63	<0.001^***^
	Genus	0.06	2.95	<0.001^***^
Orchid bees	Full model	0.2	2.8	<0.001^***^
	Overlap	0.003	–	–
	Elevation	0.15	2.57	<0.001^***^
	Genus	0.04	3.23	0.002^**^
Sweat bees	Full model	0.18	2.13	<0.001^***^
	Overlap	0.03	–	–
	Elevation	0.06	1.75	<0.001^***^
	Genus	0.09	3.16	<0.001^***^

Adjusted R^**2**^ values represent the estimated percentage of variance explained by each of the predictor variables (elevation and host) tested for each individual host tribe. Significant differences are denoted with asterisks as follows: ^*^p < 0.05, ^**^p < 0.01, and ^***^p < 0.001.

### 3.4 Partitioning of microbial beta diversity (turnover and nestedness)

Partitioning β-diversity between hosts along the elevation gradient revealed that microbial β-diversity was predominantly driven by species turnover (β_BC − bal_) for all hosts (Supplementary Table S4a). This indicates that changes in species composition rather than loss of microbial species, play a critical role in shaping microbial communities in response to elevation. Additionally, microbial turnover increased at high elevations for bumble, stingless, orchid, and sweat bees (Supplementary Table S4b), suggesting a dynamic and adaptative response of microbial taxa to environmental conditions influenced by elevation. In contrast, we found that on average, for honey bees, dissimilarity due to nestedness marginally increased with elevation (Supplementary Table S4b), indicating that higher-elevation communities consist of fewer microbial taxa that are subsets of those found at lower elevations (Baselga, 2017).

### 3.5 Effects of elevation on dominant bacterial groups in the gut microbiota

**Figure 4:**
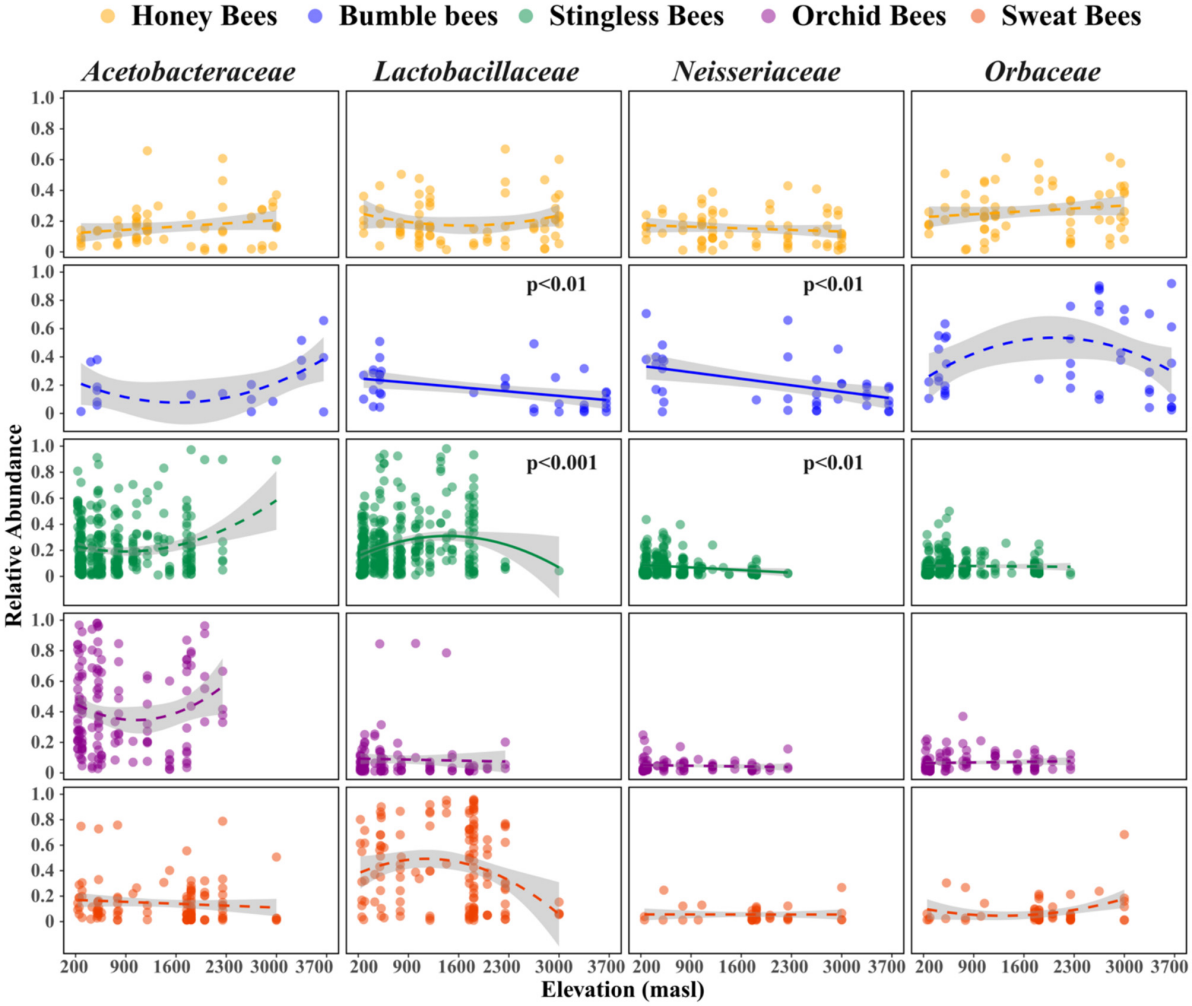
Variation in relative abundances of the major bacteria families present in the gut along the elevation gradient. Elevation significantly affects the relative abundances of *Lactobacillaceae* and *Neisseriaceae* families in bumble and stingless bees. Shown are trend lines from model with their standard deviations. Generalized linear models were used to estimate trends of elevational richness (Gaussian family). Solid trend lines along elevations represent significant differences.

The analysis of dominant bacterial families across the different bee tribes revealed distinct patterns in microbial community composition (Figure 4, Supplementary Table S5). In honey bees, no significant associations were observed for any bacterial families. In contrast, bumble bees exhibited significant declines in *Lactobacillaceae* and *Neisseriaceae*, indicating a potential shift in their gut microbiota. Stingless bees showed a significant increase in *Lactobacillaceae* and a decrease in *Neisseriaceae*, suggesting a dynamic response to environmental factors. Orchid bees and sweat bees did not demonstrate significant changes in any of the evaluated families. These findings highlight the varying influences of host taxonomic identity on gut microbial composition along different elevations, emphasizing that elevation plays a crucial role in shaping gut microbiota depending on the host species.

### 3.6 Core microbiome is only slightly influenced by elevation in stingless bees and orchid bees

In our study, we found the presence of *Snodgrassella, Gilliamella, Bifidobacterium*, and *Lactobacillus*, in four of our hosts: honey-, bumble-, stingless-, and orchid bees (Supplementary Table S6a). These taxa were absent from the sweat bees' core microbiome, which instead included *Apilactobacillus* and *Wolbachia*. LMM analyses revealed that the cumulative abundance of taxa in the core microbiome was negatively associated with elevation for stingless bees and positively for orchid bees (Supplementary Figure S3; Supplementary Table S6b). In contrast, for honey, bumble, and sweat bees, the abundance of the core microbiome was not affected by elevation (Supplementary Figure S3; Supplementary Table S6b).

## 4 Discussion

Our study reveals a significant decline in bacterial diversity and a pronounced turnover of microbial taxa along the elevation gradient, with marked differences among host tribes. Host taxonomic identity was identified as a robust predictor of gut microbial community composition, despite the notable turnover of both microbial and host taxa across the gradient. Within bumble bees and stingless bees, the observed shifts in microbial composition were predominantly attributed to environmental alterations correlating with elevation. These results underscore the substantial impact of elevation and host taxonomic identity on the gut microbiome, emphasizing their critical roles in shaping microbial diversity and community structure.

### 4.1 Microbial alpha diversity patterns along the elevation gradient

Honey-, stingless-, and orchid bees showed a decrease in microbial richness and diversity at higher altitudes. This trend could be explained by the environmental changes associated with elevation (e.g., temperature), which act as strong filtering factors against existing environmental microbial species (Sepulveda and Moeller, 2020; Yuan et al., 2021). A decrease in diversity with increasing elevation is a typical pattern for tropical mountains (Bryant et al., 2008; Hoiss et al., 2012; McCain and Grytnes, 2010; Perillo et al., 2021). This trend aligns with predictions regarding temperature limitations and the metabolic theory of ecology (Albrecht et al., 2021; Brown et al., 2004; Iltis et al., 2022), both of which indicate a decline in diversity associated with lower temperatures (McCain and Grytnes, 2010).

However, the relationship between elevation and microbial diversity is not consistent across microbial communities and hosts species (Hariprasath et al., 2025; Maihoff et al., 2025; Mayr et al., 2021; Palmer-Young et al., 2019; Sudhagar et al., 2017). In sweat bees we observed an unimodal pattern on microbial richness and general trend of microbial diversity increasing with elevation. Our findings are consistent with the patterns reported by Mayr et al. (2021) in *Lasioglossum* bees at Mt Kilimanjaro, suggesting that these wild bee microbiomes tend to be more influenced by environmental variables (Keller et al., 2013). In bumble bees, the richness of microbial taxa increased at higher elevations, a pattern that has also been reported in other studies (Maihoff et al., 2025; McCain and Grytnes, 2010). Along our elevation gradient, we observed replacement among bumble bee species inhabiting different environmental niches. These variations in host distributions reflect the distinct adaptations to changing climatic conditions at each elevation (Dong et al., 2024; Kwong et al., 2014). While it is known that some bumble bee species are capable of thriving at high elevations, there is currently limited information regarding their thermoregulatory abilities and how these may affect microbial diversity in the same environment. Bumble bee species that thrive at high elevations tend to exhibit high diversity in gut symbiont composition (Maihoff et al., 2025), a phenomenon also observed in our study. This increasing microbial diversity may be attributed to a reduction in pathogen pressure at higher elevations, which could be partially influenced by the gut microbiome (Palmer-Young et al., 2023). Consequently, the high cost of maintaining a consistent gut microbiome may be unnecessary and its loss may translate into an adaptative advantage (Maihoff et al., 2025). We further support this idea with our finding that the abundance of the core microbiome decreases with increasing elevation, likely because bumble bee foragers occasionally encounter environmental bacteria that largely displace the associated core bacterial groups (Li et al., 2015). Similar microbiota of bumble bees showed a rapid increase in diversity and reduction of core taxa when shifted from warmer indoor into colder outdoor conditions (Weinhold et al., 2024).

### 4.2 Host taxonomic identity and elevation influence the microbial community composition

We found host tribe-specific gut microbiome compositions along the elevation gradient, highlighting a strong relationship between the gut bacteria and their hosts. These findings do not account for the role of abiotic factors; however, they indicate that host identity is a more crucial factor influencing microbiome compositions. Our results can reflect distinct microbial interactions within their gut environments, influenced by various factors related to their host's ecology, including differing life histories, reproductive strategies, morphological characteristics, and seasonality (Kueneman et al., 2023). In particular, host sociality promotes the development of heritable microbial communities by creating a system that offers dependable transmission routes between host within similar environments (Kwong et al., 2017). However, hosts with lower degrees of social organization, ranging from communal to solitary, are likely to have less stable microbial communities largely acquired from the immediate environment (Voulgari-Kokota et al., 2019).

#### 4.2.1 Microbiomes are more host-specific rather than influenced by elevation

Along our gradient, host taxonomic identity seems to be more important for microbial community structure in sweat and bumble bees. This finding was particularly surprising given the pronounced microbial and host species turnover observed across our elevation gradient. While the host taxonomic identity emerged as the primary factor influencing microbial structure, it is noteworthy that sweat- and bumble bees are associated with a predominantly unique set of microbial taxa at each elevation. These distinct taxa differ from those found in congener hosts at other elevations, highlighting the complexity of microbial associations within these groups. The patterns of microbial community structure observed here could relate to the unique biotic environments provided by different host species/genera, which select specific microbial partners (Kwong et al., 2017). Thus, host selection remains a strong determinant of microbial composition regardless of local abiotic conditions in these groups of bees (Li et al., 2023).

There was no evidence that elevation is influencing the microbial compositions in honey bees, it might be due to their ability to maintain brood temperatures (Jones et al., 2004; Palmer-Young et al., 2023). This ability may allow their gut microbial symbionts to be less influenced by the environmental gradient, as we observed in our study. Moreover, the colony management of honey bees (i.e., sociality, storage of food reserves) facilitates, to some extent, compensation for several factors changing with the gradient (Hammer et al., 2021b). As a result, they manage to provide suitable conditions that are favorable for their core gut microbiota members.

#### 4.2.2 Effects of elevation on microbial community structure

The effect of elevation was notably stronger in the microbial communities associated with orchid and stingless bees than in other host tribes. It is noteworthy though that the limit in the distribution for most of the orchid and stingless bees taxa is around 2,000 masl along our gradient. Therefore, several factors could be related with the significant contribution of elevation for these hosts.

Elevation influences the community composition by incorporating diverse environmental factors (McCain and Grytnes, 2010; Wang et al., 2022). Additionally, environmental parameters change more rapidly along an elevation gradient compared to a latitudinal gradient (Margesin and Niklinska, 2019). In particular, changes in temperature have been linked to alterations in the community composition and relative abundances on bacterial taxa within individual hosts e.g., increases in temperature have been associated with increased relative abundances of Proteobacteria (Sepulveda and Moeller, 2020). Indeed, we demonstrated that microbial communities located at similar elevations tend to have more similarities in species composition. As we compare more distant communities, we observe more significant variations in the species present. Further, by partitioning the microbial beta diversity in nestedness and turnover (Ulrich and Almeida-Neto, 2012), we unveiled the differential mechanisms underlying microbial community assembly under changes in elevation (Baselga, 2010). Shifts in microbial communities were mainly driven by species replacement (i.e., turnover) rather than by spatial gain/loss of species (i.e., nestedness). These results are congruent with a meta-analysis across differing macroorganisms, where the effect of species turnover was typically the larger component of total beta diversity (Soininen et al., 2018). In this context, local environmental heterogeneity enhances species turnover, as species have varying niche requirements, allowing different species to thrive in distinct environments (Jankowski et al., 2013; Wang et al., 2018). In particular, the turnover of microbial taxa associated with bumble-, orchid-, stingless- and sweat bees was promoted at higher elevations or at the limits of their distribution, which could favor microbial species adapted to these environments (Albrecht et al., 2021). Our study confirms the key role of the environmental changes with elevation in driving microbial composition along our gradient, which is, in turn, due to the high turnover of species with narrow elevation ranges (Jankowski et al., 2013). Interestingly, despite high microbial species turnover, the contribution of microbial nestedness was significant for honey bees and increased as one approached to the top of the elevation gradient. This implies that the microbial symbionts associated with honey bees in harsher environments represent a nested subset of those found in more favorable environments (Ulrich and Almeida-Neto, 2012). Thus, environmental filtering reduces microbial richness and promotes nestedness (Jankowski et al., 2013; Wang et al., 2018). These patterns are consistent with previous reports indicating that elevation reduces species richness through local extinction (Classen et al., 2015; Hoiss et al., 2012; Perillo et al., 2021; Dzekashu et al., 2023). Our results suggest that the assembly mechanisms governing the gain or loss of species can play a more critical role in shaping the microbial composition of honey bees than species replacement in our elevation gradient.

### 4.3 Changes in relative abundances of major bacterial taxa along the elevation gradient

In our study, we found that the composition of bacterial communities was influenced by the host identity and also the elevation of the host habitat, indicating potential adaptations of gut microbiota to various ecological niches (Dong et al., 2024; Maihoff et al., 2025). This observation aligns with previous research, which has shown that species replacements of gut microbiota along elevation gradients are common due to the differing adaptations to local conditions, as noted in bumble bees (Dong et al., 2024), sweat bees (Mayr et al., 2021), and honey bees (Hariprasath et al., 2025). Interestingly, our findings revealed that *Lactobacillaceae*, the most abundant family in the gut of bees (Kueneman et al., 2023; Mayr et al., 2021), strongly increased with elevation for stingless bees but decreased at high elevations in bumble bees, suggesting host driven changes. The decline of *Lactobacillaceae* at high elevations was also reported by Mayr et al. (2021) in sweat bees. Indeed, heat tolerance experiments in bumble bees demonstrated that *Lactobacillaceae* appear to have greater abundance in the gut as temperature increases (Maihoff et al., 2025; Palmer-Young et al., 2019). It is plausible that, since stingless bees can thrive up to 2,000 m, the abundance of *Lactobacillaceae* increases until this elevation and beyond 2,000 m, this abundance is likely to decrease. However, the absence of stingless bees at higher elevations limits our ability to observe their presence and any corresponding changes in *Lactobacillaceae* abundance. We also found a decline in *Neisseriaceae* with elevation in bumble- and stingless bees. The decline in relative abundances at higher elevations suggests that these symbionts struggle to sustain growth in prolonged cold conditions. Our findings are consistent with previously observed correlations between thermotolerance of *Neisseriaceae* and the local thermal environment. Certain strains within this family are heat-resistant and tend to grow at a slower rate below 35 °C, likely due to their adaptation to the higher body temperatures maintained by bees through thermoregulation (Hammer et al., 2021a). Finally, honey-, sweat- and orchid bees gut microbial families were more resilient in response to environmental change.

While our study represents the most comprehensive survey of gut microbial communities associated with bees along a complete elevation gradient to date, it is important to acknowledge a few limitations. The absence of certain host taxa within each tribe at various elevations, combined with the limited taxonomic resolution of most host groups (typically only to genus level) did not allow us to compare full elevational gradients at host species level for each tribe. Still, our findings provide valuable insights into the dynamics of gut microbiota in relation to elevation and host identity, paving the way for future research in understanding the driver of wild bee microbiota.

## 5 Conclusions

Our study presents a detailed investigation of the diversity and community composition patterns of gut microbiota associated with five bee tribes along a complete elevation gradient in the Peruvian Andes. Exploring the interplay between abiotic and biotic factors influencing the gut microbiota of bees could provide valuable insights into the potential impacts of climate warming on host-microbe symbiosis. This opens the door to future research on how changes in the gut microbiome in response to environmental changes may affect host fitness within natural ecosystems.

## Data Availability

The datasets presented in this study can be found in online repositories. The names of the repository/repositories and accession number(s) can be found below: https://www.ncbi.nlm.nih.gov/, SUB15380081.

## References

[B1] Albrecht J. Peters M. K. Becker J. N. Behler C. Classen A. Ensslin A.. (2021). Species richness is more important for ecosystem functioning than species turnover along an elevational gradient. Nat. Ecol. Evol. 5, 1582–1593. 10.1038/s41559-021-01550-9 34545216

[B2] Allen L. Villacampa J. (2017). Orchid bees of the Manu Learning Centre, Perú/Abejas de las orquídeas del Manu Learning Centre, Perú. Manu: Crees Foundation, 22p.

[B3] Barton K. (2009). MuMIn: Multi-model Inference. R Package Version 1. 0. 0. Available online at: http://r-forge.~r-project.org/projects/mumin/ (Accessed July 21, 2025).

[B4] Baselga A. (2010). Partitioning the turnover and nestedness components of beta diversity. Global Ecol. Biogeogr. 19, 134–143. 10.1111/j.1466-8238.2009.00490.x

[B5] Baselga A. (2017). Partitioning abundance-based multiple-site dissimilarity into components: balanced variation in abundance and abundance gradients. Methods Ecol. Evol. 8, 799–808. 10.1111/2041-210X.12693

[B6] Baselga A. Orme C. D. L. (2012). betapart: an R package for the study of beta diversity. Methods Ecol. Evol. 3, 808–812. 10.1111/j.2041-210X.2012.00224.x

[B7] Bates D. M. (2010). lme4: Mixed-effects Modeling with R. Available online at: http://lme4.r-forge.r-project.org/lMMwR/lrgprt.pdf

[B8] Benson D. A. Clark K. Karsch-Mizrachi I. Lipman D. J. Ostell J. Sayers E. W. (2014). GenBank. Nucleic Acids Res. 42:D32. 10.1093/nar/gkt1030 24217914 PMC3965104

[B9] Bray J. R. Curtis J. T. (1957). An ordination of the upland forest communities of southern Wisconsin. Ecol. Monogr. 27, 326–349. 10.2307/1942268

[B10] Brown J. H. Gillooly J. F. Allen A. P. Savage V. M. West G. B. (2004). Toward a metabolic theory of ecology. Ecology 85, 1771–1789. 10.1890/03-900039965227

[B11] Bryant J. A. Lamanna C. Morlon H. Kerkhoff A. J. Enquist B. J. Green J. L. (2008). Microbes on mountainsides: contrasting elevational patterns of bacterial and plant diversity. Proc. Natl. Acad. Sci. U.S.A. 105(supplement_1), 11505–11511. 10.1073/pnas.0801920105 18695215 PMC2556412

[B12] Classen A. Peters M. K. Kindeketa W. J. Appelhans T. Eardley C. D. Gikungu M. W.. (2015). Temperature versus resource constraints: which factors determine bee diversity on Mount Kilimanjaro, Tanzania. Global Ecol. Biogeogr. 24, 642–652. 10.1111/geb.12286

[B13] Cobian G. M. Egan C. P. Amend A. S. (2019). Plant–microbe specificity varies as a function of elevation. ISME J. 13, 2778–2788. 10.1038/s41396-019-0470-4 31300724 PMC6794252

[B14] DeSantis T. Z. Hugenholtz P. Larsen N. Rojas M. Brodie E. L. Keller K.. (2006). Greengenes, a chimera-checked 16S rRNA gene database and workbench compatible with ARB. Appl. Environ. Microbiol. 72, 5069–5072. 10.1128/AEM.03006-05 16820507 PMC1489311

[B15] Dong J. H. Xu X. Ren Z. X. Zhao Y. H. Zhang Y. Chen L.. (2024). The adaptation of bumblebees to extremely high elevation associated with their gut microbiota. Msystems 9, e01219–e01223. 10.1128/msystems.01219-23 38329353 PMC10949452

[B16] Douglas A. E. (2015). Multiorganismal insects: diversity and function of resident microorganisms. Annu. Rev. Entomol. 60, 17–34. 10.1146/annurev-ento-010814-020822 25341109 PMC4465791

[B17] Dzekashu F. F. Pirk C. W. Yusuf A. A. Classen A. Kiatoko N. Steffan-Dewenter I.. (2023). Seasonal and elevational changes of plant-pollinator interaction networks in East African mountains. Ecol. Evol. 13. 10.1002/ece3.10060 37187966 PMC10175727

[B18] Dzekashu F. F. Yusuf A. A. Pirk C. W. Steffan-Dewenter I. Lattorff H. M. G. Peters M. K. (2022). Floral turnover and climate drive seasonal bee diversity along a tropical elevation gradient. Ecosphere 13. 10.1002/ecs2.3964

[B19] Edgar R. C. (2004). MUSCLE: multiple sequence alignment with high accuracy and high throughput. Nucleic Acids Res. 32, 1792–1797. 10.1093/nar/gkh340 15034147 PMC390337

[B20] Edgar R. C. (2016). SINTAX: a simple non-Bayesian taxonomy classifier for 16S and ITS sequences. bioRxiv. 10.1101/074161

[B21] Engel P. Kwong W. K. McFrederick Q. Anderson K. E. Barribeau S. M. Chandler J. A.. (2016). The bee microbiome: impact on bee health and model for evolution and ecology of host-microbe interactions. MBio 7, 10–1128. 10.1128/mBio.02164-15 27118586 PMC4850275

[B22] Fernández F. Sharkey M. J. (2006). Introducción a los Hymenoptera de la Región. Humboldt: Neotropical Sociedad Colombiana de Entomología y Universidad Nacional de Colombia.

[B23] Fierer N. McCain C. M. Meir P. Zimmermann M. Rapp J. M. Silman M. R.. (2011). Microbes do not follow the elevational diversity patterns of plants and animals. Ecology 92, 797–804. 10.1890/10-1170.1 21661542

[B24] Hall B. G. (2013). Building phylogenetic trees from molecular data with MEGA. Mol. Biol. Evol. 30, 1229–1235. 10.1093/molbev/mst012 23486614

[B25] Hammer T. J. Le E. Martin A. N. Moran N. A. (2021b). The gut microbiota of bumblebees. Insectes Soc. 1–15. 10.1007/s00040-021-00837-1 PMC8956082 35342195

[B26] Hammer T. J. Le E. Moran N. A. (2021a). Thermal niches of specialized gut symbionts: the case of social bees. Proc. R. Soc. B 288. 10.1098/rspb.2020.1480 33563119 PMC7893241

[B27] Hariprasath K. Dhanvarsha M. Mohankumar S. Sudha M. Saranya N. Saminathan V. R.. (2025). Characterization of gut microbiota in *Apis cerana* Across different altitudes in the Peninsular India. BMC Ecol. Evol. 25, 1–15. 10.1186/s12862-025-02349-z 40301729 PMC12039211

[B28] Hoiss B. Krauss J. Potts S. G. Roberts S. Steffan-Dewenter I. (2012). Altitude acts as an environmental filter on phylogenetic composition, traits and diversity in bee communities. Proc. R. Soc. B Biol. Sci. 279, 4447–4456. 10.1098/rspb.2012.1581 22933374 PMC3479805

[B29] Hoiss B. Krauss J. Steffan-Dewenter I. (2015). Interactive effects of elevation, species richness and extreme climatic events on plant–pollinator networks. Glob. Chang. Biol. 21, 4086–4097. 10.1111/gcb.12968 26332102

[B30] Holzmann K. L. Alonso-Alonso P. Correa-Carmona Y. Pinos A. Yon F. Lopera A.. (2025). Net primary productivity but not its remote-sensing proxies predict mammal diversity in Andean-Amazonian rainforests. Ecology 106. 10.1002/ecy.70059 40065608 PMC11894361

[B31] Hsieh T. C. Ma K. Chao A. (2016). iNEXT: an R package for rarefaction and extrapolation of species diversity (H ill numbers). Methods Ecol. Evol. 7, 1451–1456. 10.1111/2041-210X.12613

[B32] Iltis C. Tougeron K. Hance T. Louâpre P. Foray V. (2022). A perspective on insect–microbe holobionts facing thermal fluctuations in a climate-change context. Environ. Microbiol. 24, 18–29. 10.1111/1462-2920.15826 34713541

[B33] Jaeger B. (2017). r2glmm: computes R squared for mixed (multilevel) models. R Package Version 0.1 2, 1–12. 10.32614/CRAN.package.r2glmm

[B34] Jankowski J. E. Merkord C. L. Rios W. F. Cabrera K. G. Revilla N. S. Silman M. R. (2013). The relationship of tropical bird communities to tree species composition and vegetation structure along an Andean elevational gradient. J. Biogeogr. 40, 950–962. 10.1111/jbi.12041

[B35] Jones J. C. Myerscough M. R. Graham S. Oldroyd B. P. (2004). Honey bee nest thermoregulation: diversity promotes stability. Science 305, 402–404. 10.1126/science.1096340 15218093

[B36] Keller A. Grimmer G. Steffan- Dewenter I. (2013). Diverse microbiota identified in whole intact nest chambers of the red mason bee *Osmia bicornis* (Linnaeus 1758). PLoS ONE 8:e78296. 10.1371/journal.pone.0078296 24205188 PMC3799628

[B37] Keller A. McFrederick Q. S. Dharampal P. Steffan S. Danforth B. N. Leonhardt S. D. (2021). (More than) Hitchhikers through the network: the shared microbiome of bees and flowers. Curr. Opin. Insect Sci. 44, 8–15. 10.1016/j.cois.2020.09.007 32992041

[B38] Koch H. Abrol D. P. Li J. Schmid-Hempel P. (2013). Diversity and evolutionary patterns of bacterial gut associates of corbiculate bees. Mol. Ecol. 22, 2028–2044. 10.1111/mec.12209 23347062

[B39] König S. Krauss J. Keller A. Bofinger L. Steffan-Dewenter I. (2022). Phylogenetic relatedness of food plants reveals highest insect herbivore specialization at intermediate temperatures along a broad climatic gradient. Glob. Chang. Biol. 28, 4027–4040. 10.1111/gcb.16199 35429201

[B40] Kozich J. J. Westcott S. L. Baxter N. T. Highlander S. K. Schloss P. D. (2013). Development of a dual-index sequencing strategy and curation pipeline for analyzing amplicon sequence data on the MiSeq Illumina sequencing platform. Appl. Environ. Microbiol. 79, 5112–5120. 10.1128/AEM.01043-13 23793624 PMC3753973

[B41] Kueneman J. G. Bonadies E. Thomas D. Roubik D. W. Wcislo W. T. (2023). Neotropical bee microbiomes point to a fragmented social core and strong species-level effects. Microbiome 11:150. 10.1186/s40168-023-01593-z 37452376 PMC10347802

[B42] Kwong W. K. Engel P. Koch H. Moran N. A. (2014). Genomics and host specialization of honey bee and bumble bee gut symbionts. Proc. Nat. Acad. Sci. U.S.A. 111, 11509–11514. 10.1073/pnas.1405838111 25053814 PMC4128107

[B43] Kwong W. K. Medina L. A. Koch H. Sing K. W. Soh E. J. Y. Ascher J. S.. (2017). Dynamic microbiome evolution in social bees. Sci. Adv. 3. 10.1126/sciadv.1600513 28435856 PMC5371421

[B44] Lahti L. Shetty S. (2017). Microbiome R Package. Bioconductor.

[B45] Lara-Romero C. Seguí J. Pérez-Delgado A. Nogales M. Traveset A. (2019). Beta diversity and specialization in plant-pollinator networks along an elevational gradient. J. Biogeogr. 46, 1598–1610. 10.1111/jbi.13615

[B46] Leonhardt S. D. Peters B. Keller A. (2022). Do amino and fatty acid profiles of pollen provisions correlate with bacterial microbiomes in the mason bee *Osmia bicornis*?. Philos. Trans. R. Soc. B 377. 10.1098/rstb.2021.0171 35491605 PMC9058536

[B47] Li J. Powell J. E. Guo J. Evans J. D. Wu J. Williams P.. (2015). Two gut community enterotypes recur in diverse bumblebee species. Curr. Biol. 25, R652–R653. 10.1016/j.cub.2015.06.031 26241138

[B48] Li J. Sauers L. Zhuang D. Ren H. Guo J. Wang L.. (2023). Divergence and convergence of gut microbiomes of wild insect pollinators. MBio 14:e01270-23. 10.1128/mbio.01270-23 37504575 PMC10470603

[B49] Maihoff F. Bofinger L. Brenzinger K. Keller A. Classen A. (2025). Exploring climate-related gut microbiome variation in bumble bees: an experimental and observational perspective. Ecology 106:e70066. 10.1002/ecy.70066 40129109 PMC11933737

[B50] Mantel N. (1967). The detection of disease clustering and a generalized regression approach. Cancer Res. 27(2_Part_1), 209–220. 6018555

[B51] Margesin R. Niklinska M. A. (2019). Editorial: Elevation gradients: microbial indicators of climate change? Front. Microbiol. 10:2405. 10.3389/fmicb.2019.02405 31681250 PMC6813652

[B52] Mayr A. V. Keller A. Peters M. K. Grimmer G. Krischke B. Geyer M.. (2021). Cryptic species and hidden ecological interactions of halictine bees along an elevational gradient. Ecol. Evol. 11, 7700–7712. 10.1002/ece3.7605 34188845 PMC8216903

[B53] McCain C. M. Garfinkel C. F. (2021). Climate change and elevational range shifts in insects. Curr. Opin. Insect Sci. 47, 111–118. 10.1016/j.cois.2021.06.003 34175465

[B54] McCain C. M. Grytnes J. A. (2010). Elevational gradients in species richness. eLS. 15, 1–10. 10.1002/9780470015902.a0022548

[B55] McFrederick Q. S. Wcislo W. T. Taylor D. R. Ishak H. D. Dowd S. E. Mueller U. G. (2012). Environment or kin: whence do bees obtain acidophilic bacteria? Mol. Ecol. 21, 1754–1768. 10.1111/j.1365-294X.2012.05496.x 22340254

[B56] McMurdie P. J. Holmes S. (2013). phyloseq: an R package for reproducible interactive analysis and graphics of microbiome census data. PLoS ONE 8:e61217. 10.1371/journal.pone.0061217 23630581 PMC3632530

[B57] Mee L. Barribeau S. M. (2023). Influence of social lifestyles on host–microbe symbioses in the bees. Ecol. Evol. 13. 10.1002/ece3.10679 37928198 PMC10620586

[B58] Nakagawa S. Schielzeth H. (2013). A general and simple method for obtaining R2 from generalized linear mixed-effects models. Methods Ecol. Evol. 4, 133–142. 10.1111/j.2041-210x.2012.00261.x30239975

[B59] Nottingham A. T. Fierer N. Turner B. L. Whitaker J. Ostle N. J. McNamara N. P.. (2018). Microbes follow Humboldt: temperature drives plant and soil microbial diversity patterns from the Amazon to the Andes. Ecology 99, 2455–2466. 10.1002/ecy.2482 30076592 PMC6850070

[B60] Oksanen J. Blanchet F. G. Kindt R. Legendre P. Minchin P. R. O'hara R. B.. (2013). Package ‘vegan'. Community Ecology Package, Version 2, 1–295.

[B61] Palmer-Young E. C. Markowitz L. M. Huang W. F. Evans J. D. (2023). High temperatures augment inhibition of parasites by a honey bee gut symbiont. Appl. Environ. Microbiol. 89. 10.1128/aem.01023-23 37791764 PMC10617414

[B62] Palmer-Young E. C. Ngor L. Burciaga Nevarez R. Rothman J. A. Raffel T. R. McFrederick Q. S. (2019). Temperature dependence of parasitic infection and gut bacterial communities in bumble bees. Environ. Microbiol. 21, 4706–4723. 10.1111/1462-2920.14805 31573120 PMC7316186

[B63] Pellissier L. Albouy C. Bascompte J. Farwig N. Graham C. Loreau M.. (2018). Comparing species interaction networks along environmental gradients. Biol. Rev. 93, 785–800. 10.1111/brv.12366 28941124

[B64] Perillo L. N. Castro F. S. D. Solar R. Neves F. D. S. (2021). Disentangling the effects of latitudinal and elevational gradients on bee, wasp, and ant diversity in an ancient neotropical mountain range. J. Biogeogr. 48, 1564–1578. 10.1111/jbi.14095

[B65] Peters M. K. Hemp A. Appelhans T. Becker J. N. Behler C. Classen A.. (2019). Climate–land-use interactions shape tropical mountain biodiversity and ecosystem functions. Nature 568, 88–92. 10.1038/s41586-019-1048-z 30918402

[B66] Peters M. K. Hemp A. Appelhans T. Behler C. Classen A. Detsch F.. (2016). Predictors of elevational biodiversity gradients change from single taxa to the multi-taxa community level. Nat. Commun. 7:13736. 10.1038/ncomms13736 28004657 PMC5192166

[B67] Pitteloud C. Walser J. C. Descombes P. Novaes de Santana C. Rasmann S. Pellissier L. (2021). The structure of plant–herbivore interaction networks vary along elevational gradients in the European Alps. J. Biogeogr. 48, 465–476. 10.1111/jbi.14014

[B68] Quast C. Pruesse E. Yilmaz P. Gerken J. Schweer T. Yarza P.. (2012). The SILVA ribosomal RNA gene database project: improved data processing and web-based tools. Nucleic Acids Res. 41, D590–D596. 10.1093/nar/gks1219 23193283 PMC3531112

[B69] R Core Team (2021). R: A Language and Environment for Statistical Computing. Vienna: R Foundation for Statistical Computing.

[B70] Ramette A. Tiedje J. M. (2007). Multiscale responses of microbial life to spatial distance and environmental heterogeneity in a patchy ecosystem. Proc. Nat. Acad. Sci. U.S.A. 104, 2761–2766. 10.1073/pnas.0610671104 17296935 PMC1815255

[B71] Rasmussen C. (2003). Clave de identificación para las especies peruanas de *Bombus Latreille*, 1809 (Hymenoptera, Apidae), con notas sobre su biología y distribución. Rev. Peruana Entolomogía 43, 31–45.

[B72] Rasmussen C. Vasquez C. D. (2019). Abejas sin aguijón (Apidae: Meliponini) en Loreto, Perú. Available online at: http://repositorio.iiap.org.pe/bitstream/IIAP/396/1/Delgado_libro_2019a.pdf

[B73] Rognes T. Flouri T. Nichols B. Quince C. Mahé F. (2016). VSEARCH: a versatile open-source tool for metagenomics. PeerJ 4. 10.7717/peerj.2584 27781170 PMC5075697

[B74] Schmidt K. Engel P. (2021). Mechanisms underlying gut microbiota–host interactions in insects. J. Exp. Biol. 224. 10.1242/jeb.207696 33509844

[B75] Sepulveda J. Moeller A. H. (2020). The effects of temperature on animal gut microbiomes. Front. Microbiol. 11:384. 10.3389/fmicb.2020.00384 32210948 PMC7076155

[B76] Sickel W. Ankenbrand M. J. Grimmer G. Holzschuh A. Härtel S. Lanzen J.. (2015). Increased efficiency in identifying mixed pollen samples by meta-barcoding with a dual-indexing approach. BMC Ecol. 15, 1–9. 10.1186/s12898-015-0051-y 26194794 PMC4509727

[B77] Siles J. A. Margesin R. (2016). Abundance and diversity of bacterial, archaeal, and fungal communities along an altitudinal gradient in alpine forest soils: what are the driving factors? Microbial Ecol. 72, 207–220. 10.1007/s00248-016-0748-2 26961712 PMC4902835

[B78] Soininen J. Heino J. Wang J. (2018). A meta-analysis of nestedness and turnover components of beta diversity across organisms and ecosystems. Global Ecol. Biogeogr. 27, 96–109. 10.1111/geb.12660

[B79] Sudhagar S. Reddy P. R. Nagalakshmi G. (2017). Influence of elevation in structuring the gut bacterial communities of *Apis cerana* Fab. J. Entomol. Zool. Stud. 5, 434–440.

[B80] Thompson L. R. Sanders J. G. McDonald D. Amir A. Ladau J. Locey K. J.. (2017). A communal catalogue reveals Earth's multiscale microbial diversity. Nature 551, 457–463. 10.1038/nature24621 29088705 PMC6192678

[B81] Ulrich W. Almeida-Neto M. (2012). On the meanings of nestedness: back to the basics. Ecography 35, 865–871. 10.1111/j.1600-0587.2012.07671.x

[B82] Voulgari-Kokota A. Ankenbrand M. J. Grimmer G. Steffan-Dewenter I. Keller A. (2019). Linking pollen foraging of megachilid bees to their nest bacterial microbiota. Ecol. Evol. 9, 10788–10800. 10.1002/ece3.5599 31624582 PMC6787775

[B83] Wang J. Hu A. Meng F. Zhao W. Yang Y. Soininen J.. (2022). Embracing mountain microbiome and ecosystem functions under global change. New Phytol. 234, 1987–2002. 10.1111/nph.18051 35211983

[B84] Wang X. Wiegand T. Anderson-Teixeira K. J. Bourg N. A. Hao Z. Howe R.. (2018). Ecological drivers of spatial community dissimilarity, species replacement and species nestedness across temperate forests. Global Ecol. Biogeogr. 27, 581–592. 10.1111/geb.12719

[B85] Weinhold A. Grüner E. Keller A. (2024). Bumble bee microbiota shows temporal succession and increase of lactic acid bacteria when exposed to outdoor environments. Front. Cell. Infect. Microbiol. 14:1342781. 10.3389/fcimb.2024.1342781 38500505 PMC10945022

[B86] Yamaoka K. Nakagawa T. Uno T. (1978). Application of Akaike's information criterion (AIC) in the evaluation of linear pharmacokinetic equations. J. Pharmacokinet. Biopharm. 6, 165–175. 10.1007/BF01117450 671222

[B87] Yuan M. M. Guo X. Wu L. Zhang Y. A. Xiao N. Ning D.. (2021). Climate warming enhances microbial network complexity and stability. Nat. Clim. Chang. 11, 343–348. 10.1038/s41558-021-00989-9

[B88] Zheng H. Steele M. I. Leonard S. P. Motta E. V. Moran N. A. (2018). Honey bees as models for gut microbiota research. Lab Anim. 47, 317–325. 10.1038/s41684-018-0173-x 30353179 PMC6478020

