# Supplementary material for: Host identity, more than elevation, shapes bee microbiomes along a tropical elevation gradient
Source: Front Microbiol. 2025 Sep 18;16:1671348. doi: 10.3389/fmicb.2025.1671348 (PMC12488565; doi:10.3389/fmicb.2025.1671348)
Supplement: Supplementary file 1 [file Data_Sheet_1.pdf]

Supplementary Material

Supplementary figures

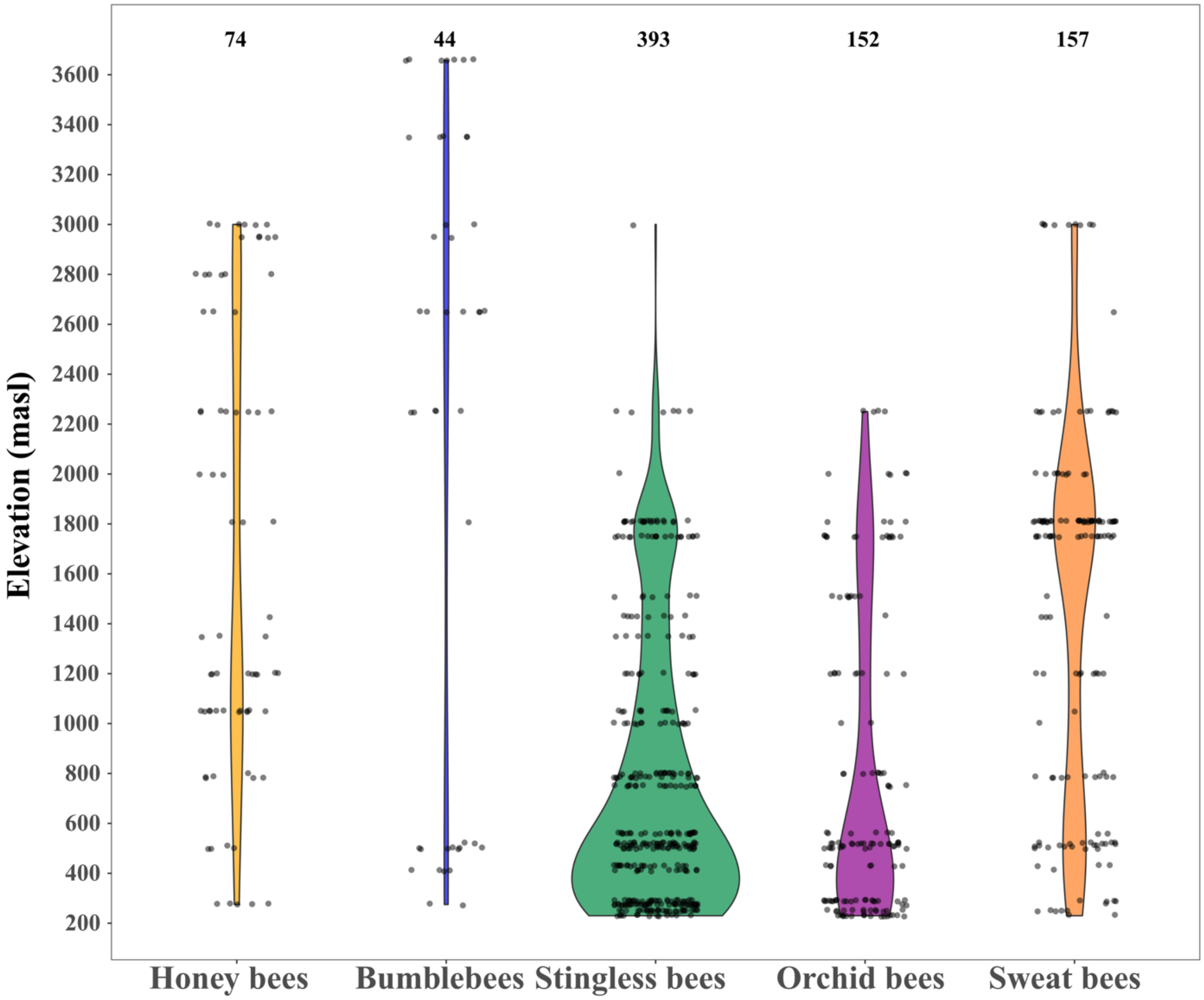

Supplementary Figure 1. Violin plots illustrate the distribution of the five bee host tribes collected along the elevation gradient. From left to right, honey bees, bumble bees species, stingless bees, orchid bees and sweat bees distributions. Top numbers represent the number of samples per host tribe.

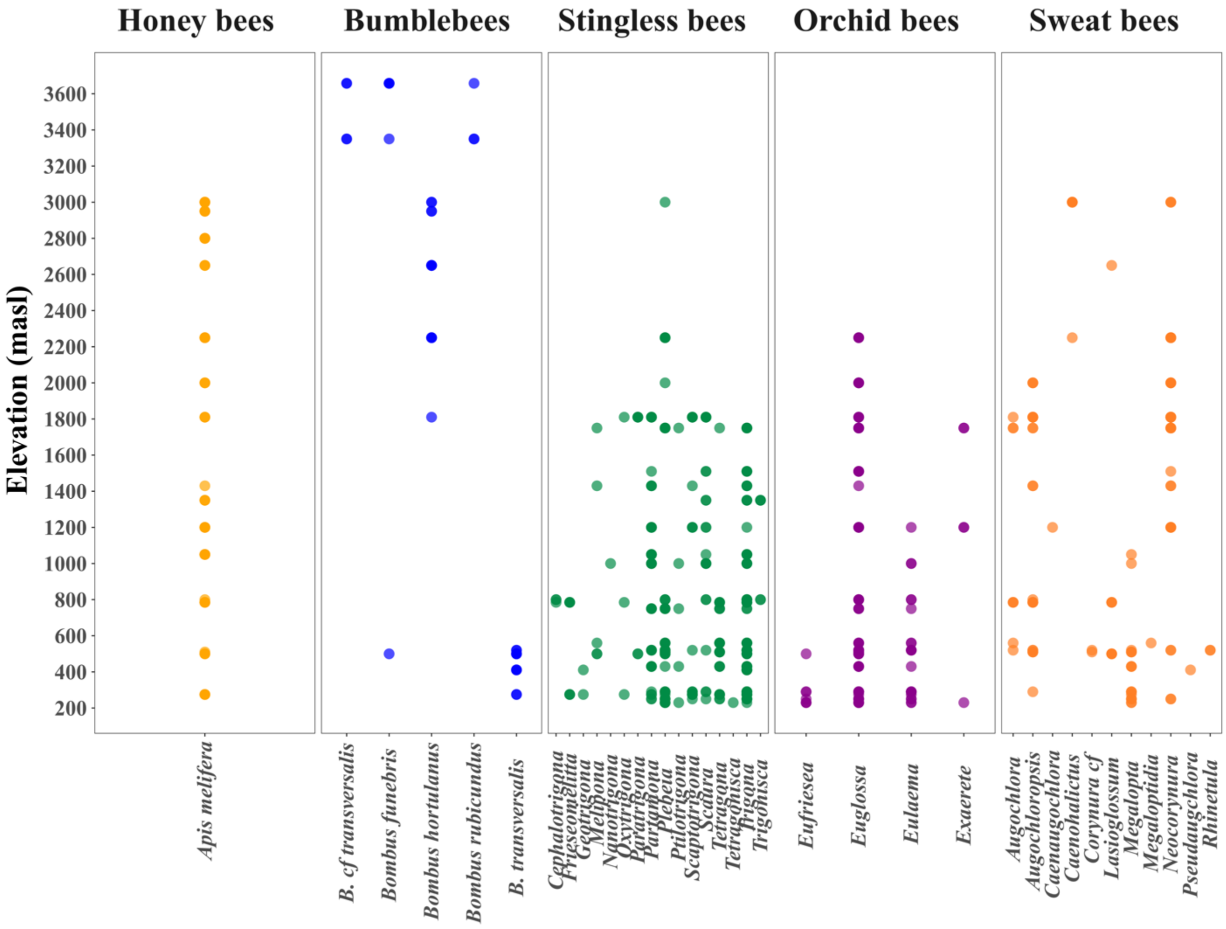

Supplementary Figure 2. Distribution of the species and genera collected for each bee tribe along the elevation gradient. One species for Apini, five species for Bombini, 16 genera for Meliponini, four genera for Euglossini and 11 genera for Halictini.

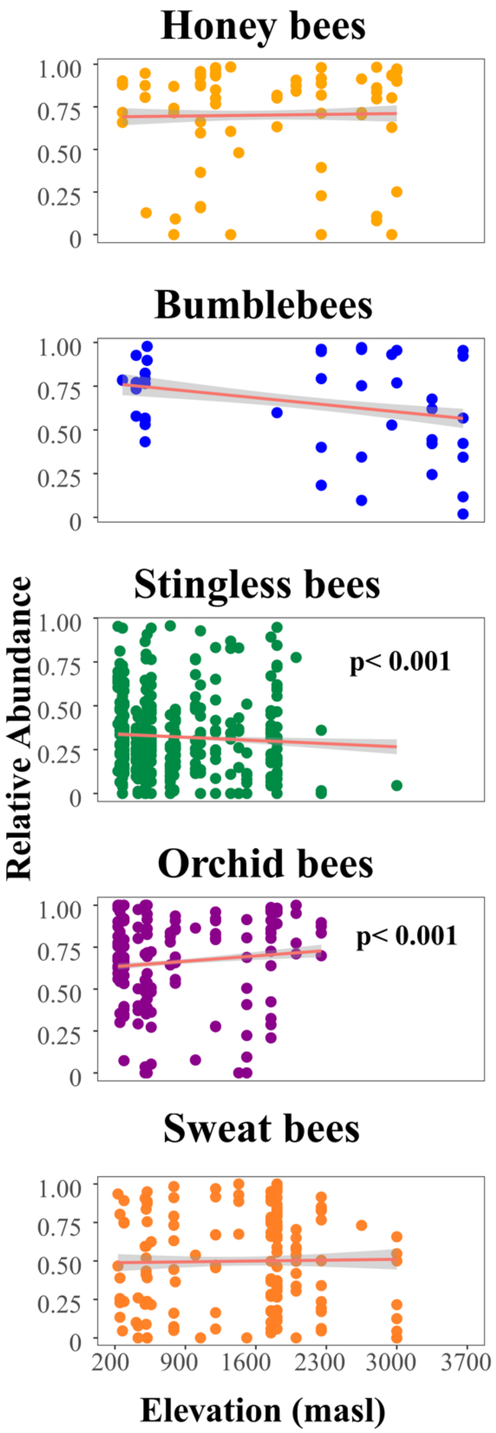

Supplementary Figure 3. Core taxa in bee gut microbiomes along the elevational gradient present at a relative abundance of above 1% and a prevalence of 50% for each host tribe**.** Core scores were plotted against elevation. Relative abundance of core taxa decreases with increasing elevation for stingless bees and increases with elevation for orchid bees. Significant differences are denoted as follows: *p < 0.05, **p < 0.01, and ***p< 0.001.

Supplementary tables

Supplementary Table 1. List of sampling locations and geographic coordinates by elevation in Perú.

Elevation (m.a.s.l)	Temperature (˚C)	Location	Plot id.	Latitude	Longitude
3750	6.7	Tres Cruces	375	-13.09608 S	-71.62952 W
3658	6.7	Tres Cruces	tc	-13.12068 S	-71.61148 W
3500	8.2	Tres Cruces	350	-13,11313 S	-71,60715 W
3350	8.2	Trocha Union/Tres Cruces	335	-13.20061 S	-71.60715 W
3250	9.3	Trocha Union/Tres Cruces	320	-13.11038 S	-71.604 64W
3000	11.1	Way	300	-13,18991 S	-71,58669 W
2950	11.2	Way	way	-13.18251 S	-71.58266 W
2800	11.2	Way	280	-13,17486 S	-71,59527 W
2650	13.0	Way	265	-13,17507 S	-71,58149W
2500	13.5	Way	250	-13.16773 S	-71.5876 W
2250	14.2	Trocha Union	224	-13.081 S	-71.566 W
2000	15.7	Trocha Union	200	-13.07377 S	-71.55938 W
1810	16.0	Trocha Union	181	-13.06972 S	-71.55544 W
1750	16.4	San Pedro	175	-13.04732 S	-71.54227 W
1510	17.8	San Pedro	151	-13.04915 S	-71.53677 W
1430	17.3	San Pedro	143	-13.06063 S	-71.5519 W
1350	18.7	San Pedro	spe	-13.05647 S	-71.54417 W
1200	18.7	Road/Santa Isabel	120	-13,03247 S	-71,52728 W
1050	19.4	Road/Quita Calzón	105	-13,02592 S	-71,49961 W
1000	20.2	Tono	100	-12.95908 S	-71.56586 W
800	21.0	Tono	080	-12.94743 S	-71.53178 W
785	21.0	Tono	to	-12.95947 S	-71.53069 W
750	23.1	Manu Station	075	-12,88754 S	-71,41399 W
560	23.1	Manu Station	056	-12,89207 S	-71,41377 W
500	23.0	Manu Station	vca	-12,89514 S	-71,40374 W
520	22.7	Manu Station	052	-12.88724 S	-71.40139 W
510	23.0	Pantiacolla	051	-12.65428 S	-71.25237 W
411	23.4	Pantiacolla	pa	-12.65584 S	-71.23175 W
430	23.6	Pantiacolla	043	-12.64186 S	-71.24106 W
290	24.2	Los Amigos	029	-12.56257 S	-70.09663 W
275	23.9	Los Amigos	la	-12.5693 S	-70.10027 W
250	23.9	Los Amigos	025	-12.55099 S	-70.09736 W
230	23.8	Los Amigos	023	-12.570795 S	-70.09245 W

Supplementary Table 2. List of all sampled bees with their elevational occurrence in meters above sea level, and their taxa classification. (a) Apini, (b) Bombini, (c) Meliponini, (d) Euglossini, and (e) Halictini.

(a) Tribe	Genus	Species	Number of individuals	Elevation	Plot id.
Apini	*Apis*	*mellifera*	5	275	Los Amigos
	Apis	*mellifera*	3	500	Villa Carmen
	Apis	*mellifera*	1	510	51
	Apis	*mellifera*	5	785	Tono
	Apis	*mellifera*	1	800	80
	Apis	*mellifera*	12	1050	105
	Apis	*mellifera*	9	1200	120
	Apis	*mellifera*	3	1350	San Pedro
	Apis	*mellifera*	1	1430	143
	Apis	*mellifera*	3	1810	181
	Apis	*mellifera*	3	2000	200
	Apis	*mellifera*	8	2250	224
	Apis	*mellifera*	3	2650	265
	Apis	*mellifera*	6	2800	280
	Apis	*mellifera*	5	2950	Wayqecha
	Apis	*mellifera*	6	3000	300

(b) Tribe	Genus	Species	Number of individuals	Elevation	Plot id.
Bombini	*Bombus*	*transversalis*	2	275	Los Amigos
	Bombus	*transversalis*	4	411	Pantiacolla
	Bombus	*transversalis*	6	500	Villa Carmen
	Bombus	*funebris*	1	500	Villa Carmen
	Bombus	*transversalis*	2	520	52
	Bombus	*hortulanus*	1	1810	181
	Bombus	*hortulanus*	5	2250	224
	Bombus	*hortulanus*	7	2650	265
	Bombus	*coccineus*	2	2950	Wayqecha
	Bombus	*hortulanus*	2	3000	300
	Bombus	*funebris*	1	3350	335
	Bombus	*rubicundus*	2	3350	335
	Bombus	*cf. transversalis*	2	3350	335
	Bombus	*rubicundus*	1	3658	Tres Cruces

	Bombus	*cf. transversalis*	2	3658	Tres Cruces
	Bombus	*funebris*	4	3658	Tres Cruces

**(c)**<br>**Tribe**	**Genus**	**Species**	**Number of individuals**	**Elevation**	**Plot id.**
	Tetragonisca		1	245	023
	Plebeia		10	245	023
	Trigona		1	245	023
	Ptilotrigona		1	245	023
	Partamona		2	250	025
	Plebeia		11	250	025
	Scaptotrigona	*postica*	1	250	025
	Scaura	*latitarsis*	1	250	025
	Tetragona		2	250	025
	Trigona		13	250	025
	Friseomelita		3	275	Los Amigos
	Geotrigona		1	275	Los Amigos
	Oxytrigona		1	275	Los Amigos
	Partamona		2	275	Los Amigos
	Plebeia		1	275	Los Amigos
	Scaptotrigona		5	275	Los Amigos
Meliponini	*Scaptotrigona*	*postica*	3	275	Los Amigos
	Tetragona		2	275	Los Amigos
	Tetragona	*perangulata*	3	275	Los Amigos
	Trigona		23	275	Los Amigos
	Trigona		14	290	029
	Partamona		1	290	029
	Plebeia		6	290	029
	Scaptotrigona		3	290	029
	Scaura	*latitarsis*	2	290	029
	Geotrigona		1	411	Pantiacolla
	Trigona		13	411	Pantiacolla
	Partamona		5	430	043
	Plebeia		1	430	043
	Ptilotrigona		1	430	043
	Tetragona		3	430	043
	Trigona		9	430	043
	Melipona		2	500	Villa Carmen

Paratrigona		2	500	Villa Carmen
Plebeia		4	500	Villa Carmen
Trigona		8	500	Villa Carmen
Plebeia		11	510	051
Tetragona		7	510	051
Partamona		6	520	052
Plebeia		7	520	052
Scaptotrigona	*postica*	1	520	052
Scaura	*atlantica*	1	520	052
Trigona		11	520	052
Melipona		1	560	056
Plebeia		5	560	056
Tetragona		6	560	056
Trigona		13	560	056
Trigona	*silvestriana*	1	560	056
Partamona		4	750	075
Plebeia		7	750	075
Ptilotrigona		1	750	075
Tetragona		3	750	075
Trigona		2	750	075
Cephalotrigona		1	785	Tono
Friseomelita		4	785	Tono
Oxytrigona		1	785	Tono
Tetratrigona		5	785	Tono
Trigona		5	785	Tono
Cephalotrigona		2	800	080
Plebeia		3	800	080
Scaura	*latitarsis*	2	800	080
Trigona		8	800	080
Trigonisca		2	800	080
Nanotrigona		1	1000	100
Partamona		5	1000	100
Ptilotrigona		1	1000	100
Scaura	*atlantica*	4	1000	100
Trigona		4	1000	100
Partamona		5	1050	105
Scaura	*atlantica*	1	1050	105

Trigona		5	1050	105
Trigona	*amazonensis*	1	1050	105
Partamona		5	1200	120
Scaptotrigona		3	1200	120
Scaura	*atlantica*	2	1200	120
Trigona		1	1200	120
Scaura	*atlantica*	2	1350	San Pedro
Trigona		3	1350	San Pedro
Trigonisca		3	1350	San Pedro
Melipona		1	1430	143
Partamona		3	1430	143
Scaptotrigona		1	1430	143
Trigona		3	1430	143
Partamona		1	1510	151
Scaura	*atlantica*	2	1510	151
Trigona		4	1510	151
Melipona		1	1750	175
Plebeia		8	1750	175
Plebeia	*frontalis*	1	1750	175
Ptilotrigona		1	1750	175
Tetragona		1	1750	175
Trigona		8	1750	175
Oxytrigona		1	1810	181
Paratrigona		5	1810	181
Partamona		5	1810	181
Scaptotrigona		5	1810	181
Scaura	*atlantica*	6	1810	181
Plebeia		1	2000	200
Plebeia		6	2250	224
Plebeia		1	3000	300

**(d)**<br>**Tribe**	**Genus**	**Number of individuals**	**Elevation**	**Plot id.**
Euglossini	*Eufrisea*	3	245	023
	Euglossa	10	245	023
	Eulaema	2	245	023
	Exaerete	1	245	023
	Eufrisea	1	250	025

Euglossa	10	250	025
Eulaema	2	250	025
Euglossa	1	275	Los Amigos
Eulaema	1	275	Los Amigos
Eufrisea	2	290	029
Euglossa	14	290	029
Eulaema	5	290	029
Euglossa	6	430	043
Eulaema	1	430	043
Eufrisea	1	500	Villa Carmen
Euglossa	7	500	Villa Carmen
Euglossa	5	510	051
Euglossa	12	520	052
Eulaema	4	520	052
Euglossa	4	560	056
Eulaema	2	560	056
Euglossa	3	750	075
Eulaema	1	750	075
Euglossa	6	800	080
Eulaema	2	800	080
Eulaema	2	1000	100
Euglossa	6	1200	120
Eulaema	1	1200	120
Exaerete	2	1200	120
Euglossa	1	1430	143
Euglossa	8	1510	151
Euglossa	11	1750	175
Exaerete	2	1750	175
Euglossa	4	1810	181
Euglossa	5	2000	200
Euglossa	4	2250	224

**(e) Tribe**	**Genus**	**Number of individuals**	**Elevation**	**Plot id.**
Halictini	*Megalopta*	2	245	023
	Neocorynura	2	250	025
	Megalopta	3	250	025

Megalopta	1	275	Los Amigos
Megalopta	3	290	029
Augochloropsis	1	290	029
Pseudaugchlora	1	411	Pantiacolla
Megalopta	3	430	043
Lasioglossum	4	500	Villa Carmen
Augochloropsis	3	510	051
Corynura cf	1	510	051
Megalopta	3	510	051
Augochlora	1	520	052
Augochloropsis	2	520	052
Corynura cf	1	520	052
Megalopta	1	520	052
Neocorynura	2	520	052
Rhinetula	2	520	052
Megaloptidia	1	560	056
Augochlora	1	560	056
Augochlora	3	785	Tono
Augochloropsis	4	785	Tono
Lasioglossum	3	785	Tono
Augochloropsis	1	800	080
Megalopta	1	1000	100
Megalopta	1	1050	105
Caenaugochlora	1	1200	120
Neocorynura	8	1200	120
Neocorynura	2	1430	143
Augochloropsis	2	1430	143
Neocorynura	1	1510	151
Augochlora	2	1750	175
Augochloropsis	2	1750	175
Neocorynura	16	1750	175
Augochlora	1	1810	181
Augochloropsis	6	1810	181
Neocorynura	32	1810	181
Augochloropsis	3	2000	200
Neocorynura	7	2000	200
Neocorynura	12	2250	224

Caenohalictus	1	2250	224
Lasioglossum	1	2650	265
Caenohalictus	6	3000	300
Neocorynura	3	3000	300

Supplementary Table 3. Effect of elevation and temperature in negative binomial generalized linear models (GLMs) on microbial richness (q = 0) and microbial diversity (q = 1) between five bee host tribes. The bolded values indicate the best fit model, distinguishing between linear and quadratic relationships. Significant differences are denoted as follows: *p < 0.05, **p < 0.01, and ***p< 0.001.

**Host**	**Predictor**	**Model: linear (β1), quadratic (β2)**	**Microbial richness (q = 0)**		**Microbial diversity (q =1)**	
			***t*-Value**	***p*-Value**	***t*-Value**	***p*-Value**
Honey bees	Elevation	β1	-2.80	0.005**	-3.13	0.002**
		**β2**	**2.01**	**0.04***	**3.86**	**< 0.001***
Bumble bees		β1	3.61	< 0.001***	3.08	0.002**
		**β2**	**1.87**	**0.06.**	**3.18**	**0.001****
Stingless bees		β1	-8.12	< 0.001***	-6.11	< 0.001***
		**β2**	**2.35**	**0.02***	**2.78**	**0.006****
Orchid bees		β1	-4.43	< 0.001***	-3.27	0.001**
		**β2**	**4.14**	**< 0.001*****	**2.52**	**0.01***
Sweat bees		β1	1.25	0.21	**3.61**	**< 0.001*****
		**β2**	**-1.89**	**0.05.**	-1.24	0.21
Honey bees	Temperature	β1	2.97	0.003**	3.28	0.001**
		β2	1.66	0.1	3.53	< 0.001***
Bumble bees		β1	-3.81	< 0.001***	-3.40	< 0.001***
		β2	1.77	0.08.	3.02	0.002**
Stingless bees		β1	7.76	< 0.001***	5.78	< 0.001***
		β2	1.41	0.15	1.83	0.07.
Orchid bees		β1	4.46	< 0.001***	3.19	0.001**
		β2	4.81	< 0.001***	2.97	0.003**
Sweat bees		β1	-1.51	0.13	-3.67	< 0.001***
		β2	-1.91	0.05.	-1.11	0.27

Supplementary Table 4. Partitioned abundance- based beta (β) diversity metrics for microbial community composition within each host examined along the gradient (a) and (b) effect of elevation on the turnover and nestedness of microbial taxa within each host tribe along the gradient, examined using Mantel correlations. Significant differences are denoted as follows: *p < 0.05, **p < 0.01, and ***p< 0.001.

**(a)**	**Measure of compositional dissimilarity (beta diversity)**		
**Host**	**Turnover ($\beta_{BC\text{-}bal}$)**	**Nestedness ($\beta_{BC\text{-}gra}$)**	**Overall (β_{BC})**
All hosts	0.998	0.000	0.998
Honey bees	0.972	0.002	0.973
Bumble bees	0.959	0.003	0.96
Stingless bees	0.996	0.000	0.996
Orchid bees	0.985	0.002	0.987
Sweat bees	0.988	0.001	0.989

**(b) Host**	**Predictor**	**Turnover (βBC-bal)**		**Nestedness (βBC-gra)**	
		r	***p*-Value**	***r***	***p*-Value**
All hosts	Elevation	0.03	0.02*	-0.01	0.78
Honey bees		0.02	0.29	0.08	0.02*
Bumble bees		0.2	< 0.001***	-0.03	0.8
Stingless bees		0.04	0.02*	-0.009	0.68
Orchid bees		0.15	< 0.001***	-0.13	1
Sweat bees		0.24	< 0.001***	-0.2	1

Supplementary Table 5. This output of generalized linear models (GLMs) presents estimates, standard errors, test statistics, and p-values for each host. Variation in relative abundances at the family level with elevation within each host. Significant differences are denoted as follows: *p < 0.05, **p < 0.01, and ***p< 0.001.

Group	Family	Estimate	std.error	Statistic	p.value
Honey bees	*Acetobacteraceae*	0.0000296	0.0000198	1.5	0.139
	Lactobacillaceae	0.00000557	0.0000191	0.291	0.772
	Neisseriaceae	-0.000015	0.0000147	-1.02	0.313
	Orbaceae	0.0000267	0.0000207	1.29	0.201
Bumble bees	*Acetobacteraceae*	0.0000447	0.0000313	1.43	0.172
	Lactobacillaceae	-0.0000443	0.0000151	-2.93	0.006**
	Neisseriaceae	-0.0000657	0.0000206	-3.19	0.002**
	Orbaceae	0.0000214	0.0000336	0.638	0.527
Stingless bees	*Acetobacteraceae*	0.0000336	0.0000193	1.74	0.082
	Lactobacillaceae	0.0000778	0.0000196	3.97	0.00008***
	Neisseriaceae	-0.0000286	0.00000968	-2.95	0.003**
	Orbaceae	-4.64E-06	0.00000933	-0.497	0.620
Orchid bees	*Acetobacteraceae*	0.0000114	0.0000376	0.304	0.761
	Lactobacillaceae	-9.26E-06	0.0000228	-0.405	0.686
	Neisseriaceae	-6.66E-06	0.00000749	-0.89	0.376
	Orbaceae	0.00000614	0.0000085	0.723	0.471
Sweat bees	*Acetobacteraceae*	-0.0000214	0.00002	-1.07	0.287
	Lactobacillaceae	-0.0000514	0.000038	-1.35	0.178
	Neisseriaceae	-3.39E-07	0.0000143	-0.0238	0.981
	Orbaceae	0.0000285	0.0000192	1.48	0.143

Supplementary Table 6. Core microbiome taxa identified at the relative abundance of 1% in at least 50% of the samples separated by host tribe (a) and (b) effect of elevation in linear mixed-effects models (LMM) on the core microbiome along the gradient. Significant differences are denoted as follows: *p < 0.05, **p < 0.01, and ***p< 0.001.

**(a) Host**	**# Core taxa**	**Core members**
Honey bees	7	*Snodgrassella, Bombilactobacillus, Commensalibacte, Bifidobacterium, Frischella, Gilliamella, Lactobacillus*
Bumble bees	5	*Snodgrassella, Orbus, Bifidobacterium, Gilliamella, Lactobacillus*
Stingless bees	5	*Bombella, Snodgrassella, Bifidobacterium, Lactobacillus, Selenomonadaceae spc.*
Orchid bees	9	*Bombella, Snodgrassella, Acinetobacter, Bifidobacterium, Pseudomonas, Gilliamella, Lactobacillus, Pantoea, Asaia*
Sweat bees	3	*Bombella, Wolbachia, Apilactobacillus*

**(b)**			**Elevation**		***Random effect (host)***
**Host**	**R2m**	**R2c**	***t*-Value**	***p*-Value**	***p*-Value**
Honey bees	0.0004		0.44	0.66	
Bumble bees	0.02	0.44	1.17	0.25	< 0.001***
Stingless bees	0.009	0.39	-5.54	< 0.001***	< 0.001***
Orchid bees	0.01	0.30	5.16	< 0.001***	< 0.001***
Sweat bees	0.006	0.36	-1.39	0.17	< 0.001***
